# On the identity of a U.S. intercepted *Conotrachelus* Dejean (Coleoptera: Curculionidae) with avocado (*Persea americana*)

**DOI:** 10.3897/BDJ.6.e26362

**Published:** 2018-11-02

**Authors:** Maria Lourdes Chamorro, Maxwell V. L. Barclay

**Affiliations:** 1 U.S. Department of Agriculture, Agricultural Research Service, Systematic Entomology Laboratory c/o Smithsonian Institution, Washington, DC, United States of America U.S. Department of Agriculture, Agricultural Research Service, Systematic Entomology Laboratory c/o Smithsonian Institution Washington, DC United States of America; 2 Department of Life Sciences, Natural History Museum, London SW7 5BD, United Kingdom Department of Life Sciences, Natural History Museum London SW7 5BD United Kingdom

**Keywords:** Food security, weevils, Molytinae, avocado seed weevils, oak, natural history collections

## Abstract

**Background:**

The multimillion-dollar avocado industry is threatened by a number of serious insect pests, including at least seven species of Curculionidae. Of these, three *Conotrachelus* species are known to develop and feed on avocados: *Conotrachelus aguacatae* Barber, *Conotrachelus perseae* Barber and *C. serpentinus* (Klug); the first two are of economic importance. Recently, a series of unrecognised *Conotrachelus* was intercepted with avocado and other commodities by the USDA at various southern U.S. ports of entry. The species most closely resembled the U.S. native *Conotrachelus posticatus* Boheman. Given the threat posed by certain species of *Conotrachelus* to avocado, the identity and biology of intercepted unknown *Conotrachelus* species becomes a matter of much concern for regulators due to the potential risk posed by non-native species to local agriculture. This study aims to determine the identity, which in turn may shed light on the biology and native distribution, of possible new non-U.S.-native weevils and provide the tools necessary to distinguish amongst phenotypically similar native species.

**New information:**

Amongst the unknown *Conotrachelus* weevils intercepted with avocados at certain U.S. ports of entry is *Conotrachelus lobatus* Champion. This poorly known species resembles a commonly collected, phenotypically variable indigenous U.S. species, *Conotrachelus posticatus*, which, on occasion, is also intercepted with avocado. *Conotrachelus lobatus* has been collected, since the early 1900s until today, along a narrow corridor in the southwest Mexican states of Michoacan, Jalisco and Nayarit. Specimen label data in natural history collections suggests the presence of this species in large numbers in early July in the avocado growing region of Mexico and, based on notes from former curators, appears to breed in acorns of the Mexican endemic oak species *Quercus obtusata*. The interception of *C. posticatus* and *C. lobatus* wth avocado does not imply strict biological association, however it reveals an important pattern of a non-native species' potential for introduction and its potential vector. Understanding all aspects of an organism's biology will better equip growers, as well as regulators, with effective and well-informed management strategies. Characters are imaged and discussed in order to help distinguish some *Conotrachelus* species belonging to *Conotrachelus* group II designated by Schoof (1942). Some characters of particular importance are the shape of the metauncus; shape of the lateral margin of the elytra and presence/absence of costate first and second elytral intervals. This study includes high-resolution images of seven *Conotrachelus* species, including the known avocado pests *C. aguacatae* and *C. perseae*, as well as the first images of *C. lobatus*, *C. scoparius* Champion and *C. squamifrons* Champion. The latter three species are not USA natives and were not included in Schoof's work. This study also confirms the important role played by natural history collections in anchoring the species' name through the study of types, which allows for the linking of biological and distribution data over time. Lectotypes are herein designated for *C. lobatus* and *C. squamifrons*.

## Introduction

Avocados (*Persea americana* Mill.) are lucrative. Between the years 2015-2016, the U.S. state of California produced 401.4 million pounds (~182 million kilograms) of avocados yielding more than $412 million dollars in crop value (California Avocado Commission 2017). Avocados also generated a substantial amount of revenue for the U.S. state of Florida, calculated to be upwards of $100 million per year in 2015 (Evans and Lozano 2014). In addition, Hawaii managed to corner a sliver of the U.S. avocado market with a production, annually, of approximately 1 million pounds (~454 thousand kg) of avocados (GreenSheet California Avocado Industry News 2013). The vast majority of avocados consumed in the U.S., however, come from Mexico with approximately 1.7 billion pounds exported in 2015 to the U.S. (Hass Avocado Board 2015). Peru, Chile, Dominican Republic and New Zealand also contribute towards satisfying the high demand for avocados in the U.S. The total volume of Hass Avocados (95% of the avocado market) entering the U.S. in 2015 totalled more than 2.1 billion pounds (990 million kg), approximately 4.3 billion avocados (Hass Avocado Board 2015, Ferdman 2015), each retailing at an average of $1.00 ($0.89 conventional, $1.52 organic).

A number of insects species are considered serious pests of, and present a potential threat to, the multibillion dollar national and international avocado industry. Within Curculionidae (Coleoptera), known avocado pests include at least seven species: five species of seed weevils, *Heilipus lauri* (Boheman, 1845), *Heilipus apiatus* (Olivier, 1807), *Conotrachelus aguacatae* Barber, 1924, *C. perseae* Barber, 1919 and *C. serpentinus* (Klug, 1829) (Molytinae); one species of stem weevil, *Copturus aguacatae* Kissinger, 1957 (Conoderinae) (USDA 2012); and the recent invasive Redbay Ambrosia Beetle, *Xyleborus glabratus* Eichhoff, 1877 (Scolytinae), vector of the laurel wilt fungus, *Raffaelea lauricola* T. C. Harr (Ophistomatales: Ophiostomataceae) (Whitehead 1979, Mayfield et al. 2008, Carrillo et al. 2012, Hoddle 2014, Fraedrich et al. 2008).

In July of 2016 and 2017, the senior author received, for urgent identification from the United States Department of Agriculture (USDA) Animal and Plant Health Inspection Service (APHIS) Plant Protection and Quarantine (PPQ), more than 10 *Conotrachelus* Dejean, 1835 (Curculionidae: Molytinae) adults intercepted with various commodities, mainly avocados, at U.S. ports of entry. These specimens were not the three *Conotrachelus* species currently associated with avocados: *C. aguacatae* (Fig. 1) *C. perseae* (Fig. 2) or *C. serpentinus*. Additional *Conotrachelus* pest species include, amongst others (Salas-Araiza and Romero-Nápoles 2012, Mancera Silva et al. 2018), the plum curculio, *C. nenuphar* (Herbst, 1797), which feeds on plum, peach and apple amongst other crops (Schoof 1942; Jenkins et al. 2006) and the following known pests of guava (*Psidium guajava* L.): *Conotrachelus copalensis* Salas and Romero, 2012; *C. dimidiatus* Champion, 1904; and *C. psidii* Marshall, 1922.

*Conotrachelus* is a New World molytine weevil genus with approximately 1,200 described species (O'Brien and Wibmer 1982, Wibmer and O'Brien 1986). In a series of papers, Fiedler treated the South American species (Fiedler 1940, Fiedler 1944, Fiedler 1952, Fiedler 1954a, Fiedler 1954b) and single-handedly described more than 900 new species. Champion (1904) treated the Mexican and Central American species and Schoof (1942) studied the species of *Conotrachelus* from North Central U.S. and provided a detailed review of the taxonomic history of the genus up to that point, as well as detailed discussion on important morphological characters to distinguish the species and sexes. The majority of *Conotrachelus* species are sexually dimorphic, thus adding to the challenge of identifying species in this hyperdiverse genus. At the time of his death, Don Whitehead (USDA, ARS, SEL) was revising, describing several new species and developing a key to the U.S. species of *Conotrachelus*, which remains unpublished. The senior author is finalising Whitehead's work, since the specimens and the manuscript are in her possession.

In this study, we determine the identity of more than 10 specimens of unfamiliar *Conotrachelus* intercepted with avocado over the course of two growing seasons (2 years).

## Materials and methods

Material examined is deposited in the following institutions:

BMNH – Natural History Museum (formerly British Museum of Natural History), London, U.K.;

CWOB – Charles W. O'Brien Collection, Green Valley, Arizona, USA;

NHRS – Naturhistoriska riksmuseet, Stockhom, Sweden;

USNM – National Museum of Natural History, Washington, DC, USA.

Specimens were examined with a Zeiss Discovery v8 stereomicroscope. Multiple habitus images were taken with the Macropod (Macroscopic Solutions) at different focal distances and combined with Zerene Stacker to achieve a greater depth of field. Images of detailed morphological features and of types were taken with an Olympus PEN5 camera mounted on a Zeiss Discovery v8 and combined with Zerene Stacker. Minor editing of the final stacked images (rotation, sharpening) was achieved using Adobe Photoshop (Adobe Products). Terminology follows Schoof (1942). Images of the types of *Conotrachelus posticatus* were obtained through the kindness of Johannes Bergsten (NHRS).

Adults were compared with the male and female type specimens of *C. lobatus* Champion, *C. squamifrons* Champion and *C. scoparius* Champion housed at BMNH. Locality information on the labels was supplemented with the gazetteer by Selander and Vaurie (1962) to the Biologia Centrali-Americana (Champion 1904). Georeference data was added to allow for this data point to be recognised in currently used software and applications for mapping the distribution of species. Champion may have collected near the stated locality and not necessarily at this precise georeference point. Dating of the Biologia Centrali-Americana in the references follows Lyal (2011).

Males can be distinguished from females by the more distal insertion of the antennae on the rostrum, whereas in females, the antennae arise more proximad on the rostrum, approximately 1/5th from the apex. In weevils generally, males and females may be readily distinguished by the presence in males of a concavity on the metasternum and on the first (and sometimes second) visible abdominal ventrite. The male genitalia characters for *C. posticatus* and *C. lobatus* were examined, compared and checked against Schoof's work.

## Taxon treatments

### Conotrachelus lobatus

Champion, 1904

http://www.biodiversitylibrary.org/item/14609#page/419/mode/1up

Conotrachelus lobatus Champion, 1904 – Champion 1904: 405. Lectotype here designated. [Type locality: Mexico, Ventanas in Durango; BMNH; male, female]. – O'Brien and Wibmer 1982: 130 [annotated checklist]. – Salas-Araiza et al. 2001: 52 [regional checklist].

#### Materials

- **Type status:** Lectotype. **Occurrence:** catalogNumber: NHMUK010801255; sex: male; otherCatalogNumbers: BMNH(E)#715649; **Taxon:** scientificName: Conotrachelus lobatus Champion, 1904; **Location:** country: Mexico; stateProvince: Durango; locality: Villa Corona; verbatimLocality: Ventanas in Durango; maximumElevationInMeters: 623.63m; locationAccordingTo: Selander and Vaurie 1962; locationRemarks: "Village on the Río del Presidio 115 km west-southwest of the city of Durango, Durango, and 100km north-east of Mazatlán, Sinaloa; 2046 feet; 23°52', 105°47'."; decimalLatitude: 23.878621; decimalLongitude: -105.773658; georeferencedBy: ML Chamorro (USNM); georeferenceSources: Google Maps 2017; **Identification:** identifiedBy: George Charles Champion; dateIdentified: 1904; **Event:** eventRemarks: Höge; **Record Level:** institutionID: BMNH; institutionCode: BMNH; collectionCode: Entomology, B.C.A. Col. iv.4; ownerInstitutionCode: BMNH; basisOfRecord: PreservedSpecimen
- **Type status:** Paralectotype. **Occurrence:** catalogNumber: NHMUK010801256; sex: female; **Taxon:** scientificName: Conotrachelus lobatus Champion, 1904; **Location:** country: Mexico; stateProvince: Durango; locality: Villa Corona; verbatimLocality: Ventanas, Durango; maximumElevationInMeters: 623.63m; locationAccordingTo: Selander and Vaurie 1962; locationRemarks: "Village on the Río del Presidio 115 km west-southwest of the city of Durango, Durango, and 100km north-east of Mazatlán, Sinaloa; 2046 feet; 23°52', 105°47'."; decimalLatitude: 23.878621; decimalLongitude: -105.773658; georeferencedBy: ML Chamorro (USNM); georeferenceSources: Google Maps 2017; **Identification:** identifiedBy: George Charles Champion; dateIdentified: 1904; **Event:** eventRemarks: Höge; **Record Level:** institutionID: BMNH; institutionCode: BMNH; collectionCode: Entomology, B.C.A. Col. iv.4; ownerInstitutionCode: BMNH; basisOfRecord: PreservedSpecimen
- **Type status:** Paralectotype. **Occurrence:** catalogNumber: NHMUK010801257; sex: female; disposition: BMNH; **Taxon:** scientificName: Conotrachelus lobatus Champion, 1904; **Location:** country: Mexico; stateProvince: Durango; locality: Villa Corona; verbatimLocality: Ventanas, Durango; maximumElevationInMeters: 623.63m; locationAccordingTo: Selander and Vaurie 1962; locationRemarks: "Village on the Río del Presidio 115 km west-southwest of the city of Durango, Durango, and 100km north-east of Mazatlán, Sinaloa; 2046 feet; 23°52', 105°47'."; decimalLatitude: 23.878621; decimalLongitude: -105.773658; georeferencedBy: ML Chamorro (USNM); georeferenceSources: Google Maps 2017; **Identification:** identifiedBy: George Charles Champion; dateIdentified: 1904; **Event:** eventRemarks: Höge; **Record Level:** institutionID: BMNH; institutionCode: BMNH; collectionCode: Entomology, B.C.A. Col. iv.4; ownerInstitutionCode: BMNH; basisOfRecord: PreservedSpecimen
- **Type status:** Other material. **Occurrence:** catalogNumber: USNMENT01448049; sex: male; disposition: dissected male; **Location:** higherGeography: North America; verbatimLocality: Intercepted on Carica papaya at Pharr CBP [origin not disclosed]; **Event:** eventDate: 2016-07-07
- **Type status:** Other material. **Occurrence:** catalogNumber: USNMENT01448048; sex: male; **Location:** country: Mexico; stateProvince: Durango; locality: El Salto; decimalLatitude: 23.780885; decimalLongitude: -105.352092; georeferencedBy: ML Chamorro; georeferenceProtocol: Google maps; georeferenceRemarks: Random point within El Salto; **Identification:** identifiedBy: ML Chamorro; dateIdentified: 2018; **Event:** eventDate: 1968-07-07; eventRemarks: Collectors GHalffter, PReyes C; **Record Level:** institutionID: USNM
- **Type status:** Other material. **Occurrence:** catalogNumber: USNMENT01448045; sex: male; **Location:** country: Mexico; stateProvince: Durango; locality: El Salto; decimalLatitude: 23.780885; decimalLongitude: -105.352092; georeferencedBy: ML Chamorro; georeferenceProtocol: Google maps; georeferenceRemarks: Random point within El Salto; **Identification:** identifiedBy: ML Chamorro; dateIdentified: 2018; **Event:** eventDate: 1968-07-07; eventRemarks: Collectors GHalffter, PReyes C; **Record Level:** institutionID: USNM
- **Type status:** Other material. **Occurrence:** catalogNumber: USNMENT01448046; sex: male; **Location:** country: Mexico; stateProvince: Durango; locality: El Salto; decimalLatitude: 23.780885; decimalLongitude: -105.352092; georeferencedBy: ML Chamorro; georeferenceProtocol: Google maps; georeferenceRemarks: Random point within El Salto; **Identification:** identifiedBy: ML Chamorro; dateIdentified: 2018; **Event:** eventDate: 1968-07-07; eventRemarks: Collectors GHalffter, PReyes C; **Record Level:** institutionID: USNM
- **Type status:** Other material. **Occurrence:** catalogNumber: USNMENT01448121; sex: male; **Location:** country: Mexico; stateProvince: Durango; locality: El Salto; decimalLatitude: 23.780885; decimalLongitude: -105.352092; georeferencedBy: ML Chamorro; georeferenceProtocol: Google maps; georeferenceRemarks: Random point within El Salto; **Identification:** identifiedBy: ML Chamorro; dateIdentified: 2018; **Event:** eventDate: 1968-07-07; eventRemarks: Collectors GHalffter, PReyes C; **Record Level:** institutionID: USNM
- **Type status:** Other material. **Occurrence:** catalogNumber: USNMENT01448122; sex: male; **Location:** country: Mexico; stateProvince: Durango; locality: El Salto; decimalLatitude: 23.780885; decimalLongitude: -105.352092; georeferencedBy: ML Chamorro; georeferenceProtocol: Google maps; georeferenceRemarks: Random point within El Salto; **Identification:** identifiedBy: ML Chamorro; dateIdentified: 2018; **Event:** eventDate: 1968-07-07; eventRemarks: Collectors GHalffter, PReyes C; **Record Level:** institutionID: USNM
- **Type status:** Other material. **Occurrence:** catalogNumber: USNMENT01448123; sex: male; **Location:** country: Mexico; stateProvince: Durango; locality: El Salto; decimalLatitude: 23.780885; decimalLongitude: -105.352092; georeferencedBy: ML Chamorro; georeferenceProtocol: Google maps; georeferenceRemarks: Random point within El Salto; **Identification:** identifiedBy: ML Chamorro; dateIdentified: 2018; **Event:** eventDate: 1968-07-07; eventRemarks: Collectors GHalffter, PReyes C; **Record Level:** institutionID: USNM
- **Type status:** Other material. **Occurrence:** catalogNumber: USNMENT01448124; sex: male; **Location:** country: Mexico; stateProvince: Durango; locality: El Salto; decimalLatitude: 23.780885; decimalLongitude: -105.352092; georeferencedBy: ML Chamorro; georeferenceProtocol: Google maps; georeferenceRemarks: Random point within El Salto; **Identification:** identifiedBy: ML Chamorro; dateIdentified: 2018; **Event:** eventDate: 1968-07-07; eventRemarks: Collectors GHalffter, PReyes C; **Record Level:** institutionID: USNM
- **Type status:** Other material. **Occurrence:** catalogNumber: USNMENT01448125; sex: male; **Location:** country: Mexico; stateProvince: Durango; locality: El Salto; decimalLatitude: 23.780885; decimalLongitude: -105.352092; georeferencedBy: ML Chamorro; georeferenceProtocol: Google maps; georeferenceRemarks: Random point within El Salto; **Identification:** identifiedBy: ML Chamorro; dateIdentified: 2018; **Event:** eventDate: 1968-07-07; eventRemarks: Collectors GHalffter, PReyes C; **Record Level:** institutionID: USNM
- **Type status:** Other material. **Occurrence:** catalogNumber: USNMENT01448126; sex: male; **Location:** country: Mexico; stateProvince: Durango; locality: El Salto; decimalLatitude: 23.780885; decimalLongitude: -105.352092; georeferencedBy: ML Chamorro; georeferenceProtocol: Google maps; georeferenceRemarks: Random point within El Salto; **Identification:** identifiedBy: ML Chamorro; dateIdentified: 2018; **Event:** eventDate: 1968-07-07; eventRemarks: Collectors GHalffter, PReyes C; **Record Level:** institutionID: USNM
- **Type status:** Other material. **Occurrence:** catalogNumber: USNMENT01448120; sex: Male; **Location:** country: Mexico; stateProvince: Jalisco; locality: La Barca; decimalLatitude: 20.285789; decimalLongitude: -102.533097; georeferencedBy: ML Chamorro; georeferenceProtocol: Google Maps; georeferenceRemarks: Random locality with La Barca; **Identification:** identifiedBy: ML Chamorro; dateIdentified: 2018; **Event:** eventDate: 1969-07-14; eventRemarks: Collector PReyesCastillo
- **Type status:** Other material. **Occurrence:** catalogNumber: USNMENT01448119; sex: Male; **Location:** country: Mexico; stateProvince: Jalisco; locality: La Barca; decimalLatitude: 20.285789; decimalLongitude: -102.533097; georeferencedBy: ML Chamorro; georeferenceProtocol: Google Maps; georeferenceRemarks: Random locality with La Barca; **Identification:** identifiedBy: ML Chamorro; dateIdentified: 2018; **Event:** eventDate: 1969-07-14; eventRemarks: Collector PReyesCastillo
- **Type status:** Other material. **Occurrence:** catalogNumber: USNMENT01448118; sex: Male; **Location:** country: Mexico; stateProvince: Michoacan; locality: Periban; decimalLatitude: 19.546127; decimalLongitude: -102.452905; georeferencedBy: ML Chamorro; georeferenceRemarks: Random point in Periba; **Event:** eventDate: 1973-07; eventRemarks: Avocado Pest Survey; Jarred from tree; Burgess Brownsville, TX
- **Type status:** Other material. **Occurrence:** catalogNumber: USNMENT01448117; sex: Male; **Location:** country: Mexico; stateProvince: Michoacan; locality: Periban; decimalLatitude: 19.546127; decimalLongitude: -102.452905; georeferencedBy: ML Chamorro; georeferenceRemarks: Random point in Periba; **Event:** eventDate: 1973-07; eventRemarks: Avocado Pest Survey; Jarred from tree; Burgess Brownsville, TX
- **Type status:** Other material. **Occurrence:** catalogNumber: USNMENT01448116; sex: Male; **Location:** country: Mexico; stateProvince: Michoacan; locality: Tingüindin [Tinquindin]; decimalLatitude: 19.733999; decimalLongitude: -102.486135; georeferencedBy: ML Chamorro; georeferenceRemarks: Random point in Tingüindin; **Event:** eventDate: 1973-07; eventRemarks: Avocado Pest Survey; Jarred from tree; Burgess Brownsville, TX
- **Type status:** Other material. **Occurrence:** catalogNumber: USNMENT01448115; sex: Male; **Location:** country: Mexico; stateProvince: Michoacan; locality: Tingüindin [Tinquindin]; decimalLatitude: 19.733999; decimalLongitude: -102.486135; georeferencedBy: ML Chamorro; georeferenceRemarks: Random point in Tingüindin; **Event:** eventDate: 1973-07; eventRemarks: Avocado Pest Survey; Jarred from tree; Burgess Brownsville, TX
- **Type status:** Other material. **Occurrence:** catalogNumber: USNMENT01448114; sex: Male; establishmentMeans: "Spm. from Michoacan, Jicalan, 3 mi. s. Uruapan (Gibson) was reared from acorns Quercus obtusata" DR Whitehead; **Location:** country: Mexico; stateProvince: Michoacan; locality: Uruapan; locationRemarks: Based on the a by D. Whitehead in the collection, this location is presumed to be in Jicalan, 3mi s. Uruapan.; decimalLatitude: 19.390149; decimalLongitude: -102.070682; georeferencedBy: ML Chamorro; georeferenceSources: Google Maps; **Event:** eventDate: 1973-09; eventRemarks: Avocado Pest Survey, Jarred from tree, Burgess, Brownsville, TX
- **Type status:** Other material. **Occurrence:** catalogNumber: USNMENT01448113; sex: Male; **Location:** country: Mexico; stateProvince: Nayarit; locality: Tepic; decimalLatitude: 21.486916; decimalLongitude: -104.829700; georeferencedBy: ML Chamorro; georeferenceSources: Google Maps; georeferenceRemarks: Random location within Tepic chosen for georeference; **Event:** samplingProtocol: light; eventDate: 1955-07-20; eventRemarks: Collected by RB & JM Selander; JMKingsolcer Collection 196
- **Type status:** Other material. **Occurrence:** catalogNumber: USNMENT01173425; **Location:** locationRemarks: Intercepted at Laredo, TX by CBP-APHIS/PPQ from cargo originating south of the border; **Event:** samplingProtocol: Inspection of cargo; eventDate: 2016-07-05; **Record Level:** informationWithheld: Origin withheld
- **Type status:** Other material. **Occurrence:** catalogNumber: USNMENT01448049; occurrenceRemarks: Ex. Carica papaya; sex: Male; previousIdentifications: Conotrachelus nr posticatus; **Location:** locality: Intercepted by Pharr CBP-APHIS/PPQ on cargo; **Event:** samplingProtocol: Insepction of cargo.; eventDate: 2016-07-07; **Record Level:** informationWithheld: Origin withheld

#### Diagnosis

*Conotrachelus lobatus* (*Figs 3, 4, 5, 6, 7b*) belongs to *Conotrachelus* Group II (*Schoof 1942*). This group of eight species was characterised by Schoof (1942: 95) based on "the presence of one femoral tooth, but a second feeble tooth (denticle) is usually present in [*C.*] *cribicollis* (Say); relative length of first and second funicular segments of antennae variable; prothorax wider than long, longitudinally carinate or not carinate; mesoscutellum from lateral aspect abruptly declivent basally; elytral intervals 3, 5, 7, 9 acutely costate, feebly convex or flattened; costae, when present, usually complete, intervals never with abrupt elytral elevations; vestiture of recumbent setae, scales or scale-like setae; suberect to erect setae usually present on elytral intervals and sometimes in prothoracic punctures; recumbent setae not condensed in a broad postmedian band; metasternum in males never grooved from meso-to metacoxa; male sometimes with dentiform metaunci; aedeagus with a dorsal membrane (except in [C.] *crataegi* Walsh), frequently with an apical process and with the transfer apparatus a complex of sclerotised bars.".

Of the species from North America north of Mexico, *Conotrachelus lobatus* is most similar to *C. posticatus* Boheman, *C. carinifer* Casey and *C. naso* LeConte. These species all have a longitudinal median prothoracic carina (Figs 3b, 4b, 11b); mesosternum with anterolateral angles truncate and prominent. *Conotrachelus naso*, however, can easily be distinguished from other members of this group by the absence of a profemoral tooth (e.g. presence: Figs 3e, 4b). Size 5.5 mm. *Conotrachelus carinifer* is distinguished by the presence on the pronotum of cavernous, punctations with raised, thin edges (Fig. 11).

*Conotrachelus lobatus* resembles *C. posticatus* in the presence of white to fulvous setae concentrated in a few scattered patches, but mainly in a narrow, posteriorly curved, postmedian elytral band (on the declivity) (Figs 3b, 4b, 7).

Male *Conotrachelus lobatus*, which have elongate fulvous setae ventrally on the fore femur and insertion of antennae subapically on the rostrum (Fig. 3a, c, d), can immediately be distinguished from other male *Conotrachelus* here treated, by the presence of a well-developed, spatulate metauncus at the distal apex of the hind tibia (Fig. 3e). *Conotrachelus lobatus* females do not posses a spatulate metauncus, instead females have the typical apically narrowing uncus (Figs 4a, b, 7b). *Conotrachelus scoparius*, instead of a spatulate metauncus, has a fulvous aggregation of elongate setae apically on the hind tibia. Male *C. posticatus* have a dentiform metauncus and are readily distinguished from other *Conotrachelus* species by having the first and second elytral intervals (interstices) costate anterior to the apical declivity (Fig. 9a, b). On the other hand, female *C. posticatus* have the first interval costate, yet usually less prominent than in the male, (Figs 7a, 10b).

Sexual dimorphism is minimal in *C. lobatus* compared to *C. posticatus*. As in the majority of *Conotrachelus*, the rostrum in the female is longer, the antennae are located more proximad and the sternum is not concave. *Conotrachelus* females are more challenging than the males to distinguish.

#### Distribution

Mexico

### Conotrachelus posticatus

Boheman in Schoenherr, 1837

http://www.biodiversitylibrary.org/item/14609#page/417/mode/1up

https://archive.org/stream/americanentomolo01sayt#page/284/mode/2up/search/posticatus

https://www.biodiversitylibrary.org/item/24769#page/418/mode/1up

Conotrachelus posticatus Say, 1831 in Say 1831:19 [nomen nudum]; Boh. in Schoenherr 1837: 406 [original description]; Champion 1904: 403 [redescription, variation, distribution]; O'Brien and Wibmer 1982: 131 [annotated checklist].

#### Diagnosis

**Figure 8.**
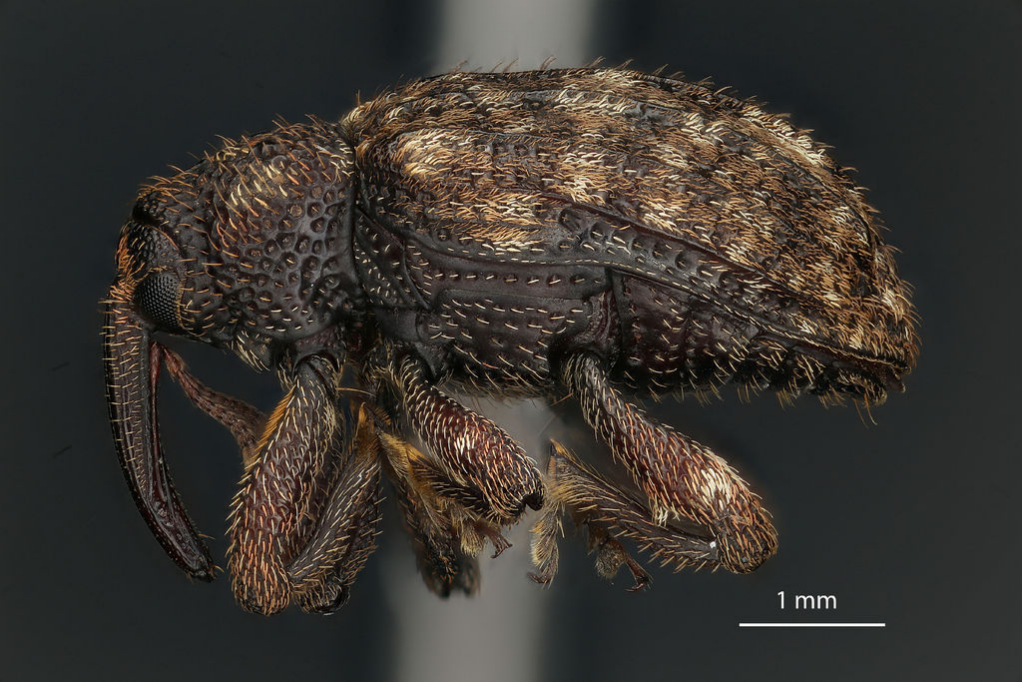
Conotrachelus nr posticatus (USNMENT01173426), female, lateral view.

One of the salient features of *C. posticatus* (Figs 7a, 8, 9, 10) is the presence of a costate first elytral interval (interstriae) in both male and females (Figs 9b, 10b, Figs 12b, 13b). In addition, males usually have the second interval costate (Fig. 9b). *Conotrachelus posticatus* also has subparallel elytral margins beyond midline, then abruptily converging (abruptily constricted in some specimens) to the apex. In other species herein treated, the elytral margins gradually converge towards the apex, hence *C. posticatus* has a rather pseudo-quadrate gestalt with the elytra roughly 2.5 times the pronotal length (Figs 7, 9b, 10b).

#### Distribution

Canada, Guatemala, Mexico, Panamá, U.S.A.

#### Notes

It is not uncommon in collections to find *Conotrachelus naso* misidentified as *C. posticatus*. The fore-femora of *C. posticatus* are distally excavate ventrally and bear a tooth, while in *C. naso*, the ventral margin of the fore-femora is largely entire. During the course of this study, we came across multiple species that resemble *C. posticatus* (e.g. elytral maculation, males with metauncus dentiform and first and second striae costate); however, upon dissection of males, differences in the median lobe confirmed these to be different species. Careful examination of the metauncus reveals additional diagnostic characters. While many of these superficially similar *C. posticatus* males possess a dentiform metauncus, slight differences exist, such as the presence of tufts of setae on the denticles themselves. Other species, in what is here termed the *C. posticatus* complex, share many characteristics with *C. naso*; for example, the ventral margins of the fore-femora are entire with either a small spine or altogether lacking one. In addition, the shape of the apex of the median lobe resembles that of *C. naso*. A revision of *C. posticatus* and related species is needed.

### Conotrachelus scoparius

Champion, 1904

http://www.biodiversitylibrary.org/item/14609#page/419/mode/1up

Conotrachelus scoparius Champion, 1904; – Champion 1904: 405 [Type locality: Mexico, Yautepec in Morelos; BMNH; male]. – O'Brien and Wibmer 1982: 132 [annotated checklist]. – Salas-Araiza et al. 2001: 52 [regional checklist].

#### Materials

- **Type status:** Holotype. **Occurrence:** catalogNumber: NHMUK010801258; sex: male; otherCatalogNumbers: BMNH(E)#71565; **Location:** country: Mexico; stateProvince: Morelos; locality: Yautepec; verbatimLocality: Yautepec, Morelos; maximumElevationInMeters: 1158m; locationAccordingTo: Selander and Vaurie 1962; locationRemarks: City east of Cuernavaca, 3800ft; 18°53', 99°04'; decimalLatitude: 18.88333; decimalLongitude: -99.06667; georeferencedBy: M.L. Chamorro (USNM); georeferenceSources: Google Maps and Selander and Vaurie 1962; **Identification:** identifiedBy: Champion; dateIdentified: 1904; **Record Level:** institutionID: BMNH; collectionCode: Entomology, B.C.A. Col. iv. 4; ownerInstitutionCode: BMNH

#### Diagnosis

Size: 4.5 mm. Currently, only the male is known for this species, which can be distinguished from other species included in this study by the presence of elongate, fulvous setae apically on all tibiae (Fig. 14).

#### Distribution

Mexico

### Conotrachelus squamifrons

Champion, 1904

http://www.biodiversitylibrary.org/item/14609#page/417/mode/1up

Conotrachelus squamifrons Champion, 1904; – Champion 1904: 403. Lectotype here designated. [Type locality: Guatemala, Zapote; BMNH; male]. – O'Brien and Wibmer 1982: 132 [annotated checklist]. – Salas-Araiza et al. 2001: 52 [regional checklist].

#### Materials

- **Type status:** Lectotype. **Occurrence:** catalogNumber: NHMUK010801259; sex: male; otherCatalogNumbers: BMNH(E)#715654; **Taxon:** scientificName: Conotrachelus squamifrons Champion, 1904; **Location:** country: Guatemala; stateProvince: Escuintla; locality: El Zapote; verbatimLocality: Zapote, Guatemala; maximumElevationInMeters: 609.6m; locationAccordingTo: Selander and Vaurie 1962; locationRemarks: Settlement about 12km northwest of Escuintla and south of Volcán de Fuego; 2000±; 14°23', 90°52'. The settlement was formerly considered to be in the department of Sacatepéquez.; decimalLatitude: 14.83333; decimalLongitude: -90.86667; georeferencedBy: ML Chamorro; georeferenceSources: Selander and Vaurie 1962; **Identification:** identifiedBy: George Charles Champion; dateIdentified: 1904; **Event:** eventRemarks: Champion; **Record Level:** institutionID: BMNH; basisOfRecord: PreservedSpecimen
- **Type status:** Paralectotype. **Occurrence:** catalogNumber: NHMUK010801262; sex: male; **Taxon:** scientificName: Conotrachelus squamifrons Champion, 1904; **Location:** country: Guatemala; locality: Zapote; verbatimLocality: Zapote, Guatemala; locationAccordingTo: Selander and Vaurie 1962; locationRemarks: Settlement about 12 km northwest of Escuintla and south of Volcán de Fuego; 2000±; 14°23', 90°52'. The settlement was formerly considered to be in the department of Sacatepéquez; decimalLatitude: 14.83333; decimalLongitude: -90.86667; georeferencedBy: M.L. Chamorro; georeferenceSources: Selander and Vaurie 1962; **Identification:** identifiedBy: George Charles Champion; dateIdentified: 1904; **Event:** eventRemarks: Champion; **Record Level:** rightsHolder: British Museum of Natural History; institutionID: BMNH; basisOfRecord: PreservedSpecimen
- **Type status:** Paralectotype. **Occurrence:** catalogNumber: NHMUK010801263; sex: female; **Taxon:** scientificName: Conotrachelus squamifrons Champion, 1904; **Location:** country: Guatemala; locality: Zapote; verbatimLocality: Zapote, Guatemala; locationAccordingTo: Selander and Vaurie 1962; locationRemarks: Settlement about 12 km northwest of Escuintla and south of Volcán de Fuego; 2000±; 14°23', 90°52'. The settlement was formerly considered to be in the department of Sacatepéquez; decimalLatitude: 14.83333; decimalLongitude: -90.86667; georeferencedBy: M.L. Chamorro; georeferenceSources: Selander and Vaurie 196; **Identification:** identifiedBy: George Charles Champion; dateIdentified: 1904; **Event:** eventRemarks: Champion; **Record Level:** institutionID: BMNH; ownerInstitutionCode: BMNH; basisOfRecord: PreservedSpecimen
- **Type status:** Paralectotype. **Occurrence:** catalogNumber: NHMUK010801264; sex: female; **Taxon:** scientificName: Conotrachelus squamifrons Champion, 1904; **Location:** country: Guatemala; locality: Zapote; verbatimLocality: Zapote, Guatemala; locationAccordingTo: Selander and Vaurie 1962; locationRemarks: Settlement about 12 km northwest of Escuintla and south of Volcán de Fuego; 2000±; 14°23', 90°52'. The settlement was formerly considered to be in the department of Sacatepéquez; decimalLatitude: 14.83333; decimalLongitude: -90.86667; georeferencedBy: M.L. Chamorro; **Identification:** identifiedBy: George Charles Champion; dateIdentified: 1904; **Event:** eventRemarks: Champion; **Record Level:** rightsHolder: BMNH; institutionID: BMNH; basisOfRecord: PreservedSpecimen
- **Type status:** Paralectotype. **Occurrence:** catalogNumber: NHMUK010801265; sex: male; **Taxon:** scientificName: Conotrachelus squamifrons Champion, 1904; **Location:** country: Guatemala; locality: Zapote; verbatimLocality: Zapote, Guatemala; locationAccordingTo: Selander and Vaurie 1962; locationRemarks: Settlement about 12 km northwest of Escuintla and south of Volcán de Fuego; 2000±; 14°23', 90°52'. The settlement was formerly considered to be in the department of Sacatepéquez; decimalLatitude: 14.83333; decimalLongitude: -90.86667; georeferencedBy: M.L. Chamorro; georeferenceSources: Selander and Vaurie 1962; **Identification:** identifiedBy: George Charles Champion; dateIdentified: 1904; **Event:** eventRemarks: Champion; **Record Level:** institutionID: BMNH; institutionCode: BMNH; basisOfRecord: PreservedSpecimen
- **Type status:** Paralectotype. **Occurrence:** catalogNumber: NHMUK010801266; sex: male; **Taxon:** scientificName: Conotrachelus squamifrons Champion, 1904; **Location:** country: Guatemala; locality: Zapote; verbatimLocality: Zapote, Guatemala; maximumElevationInMeters: 609.6 m; locationAccordingTo: Selander and Vaurie 1962; locationRemarks: Settlement about 12 km northwest of Escuintla and south of Volcán de Fuego; 2000±; 14°23', 90°52'. The settlement was formerly considered to be in the department of Sacatepéquez; decimalLatitude: 14.83333; decimalLongitude: -90.86667; georeferencedBy: ML Chamorro; georeferenceSources: Selander and Vaurie 1962; **Identification:** identifiedBy: George Charles Champion; dateIdentified: 1904; **Event:** eventRemarks: Champion; **Record Level:** institutionID: BMNH; basisOfRecord: PreservedSpecimen
- **Type status:** Paralectotype. **Occurrence:** catalogNumber: NHMUK01801267; sex: female; **Taxon:** scientificName: Conotrachelus squamifrons Champion, 1904; **Location:** country: Guatemala; locality: Cerro Zunil; verbatimLocality: Cerro Zunil 1000m (first number illegible); maximumElevationInMeters: 1219; locationAccordingTo: Selander and Vaurie 1962; locationRemarks: Volcán Zunil, Quezaltenango, Guatemala. Volcanic mountain on the Pacific slope about 12 km. southeast of Quezaltenango; 14°43', 91°29'; decimalLatitude: 14.708716; decimalLongitude: -91.48197; **Identification:** identifiedBy: George Charles Champion; dateIdentified: 1904; **Event:** eventRemarks: Champion; **Record Level:** institutionID: BMNH; basisOfRecord: PreservedSpecimen
- **Type status:** Paralectotype. **Occurrence:** catalogNumber: NHMUK010801268; sex: female; **Taxon:** scientificName: Conotrachelus squamifrons Champion, 1904; **Location:** country: Panama; locality: Bugaba, 800-1,500 ft; minimumElevationInMeters: 243; maximumElevationInMeters: 457; locationAccordingTo: Selander and Vaurie 1962; locationRemarks: Settlement on the Pacific slope about 22 km. northwest of David; 1000 feet; 8°28', 82°38'. The locality is sometimes cited erroneously as in Nicaragua.; decimalLatitude: 8.466497; decimalLongitude: -82.633333; georeferencedBy: M.L. Chamorro; **Identification:** identifiedBy: George Charles Champion; dateIdentified: 1904; **Event:** eventRemarks: Champion; **Record Level:** institutionID: BMNH; basisOfRecord: PreservedSpecimen
- **Type status:** Paralectotype. **Occurrence:** catalogNumber: NHMUK010801269; sex: male; **Taxon:** scientificName: Conotrachelus squamifrons Champion, 1904; **Location:** country: Panama; locality: Bugaba, Panama; minimumElevationInMeters: 243; maximumElevationInMeters: 457; locationAccordingTo: Selander and Vaurie 1962; locationRemarks: Bugaba, Chiriquí, Panamá. Settlement on the Pacific slope about 22 km. northwest of David; 1000 feet; 8°28', 82°38'. The locality is sometimes cited erroneously as in Nicaragua.; decimalLatitude: 8.466635; decimalLongitude: -82.633333; georeferencedBy: M.L. Chamorro; **Identification:** identifiedBy: George Charles Champion; dateIdentified: 1904; **Event:** eventRemarks: Champion; **Record Level:** institutionID: BMNH; basisOfRecord: PreservedSpecimen

#### Diagnosis

Male *C. squamifrons* (Fig. 15) can be readily distinguished by the presence of an elongate, apically narrowing dentiform metauncus, visible when viewing the tibia ventrally, dorsally and anteriorly (Fig. 15b). This metauncus may be obscured when viewing the leg laterally (Fig. 15d). In addition, *C. squamifrons* have deep pronotal punctations and the elytral surface is largely devoid of short pubescence (Fig. 15b). *Conotrachelus posticatus* males also possess a dentiform metauncus, however, the elytral characters, as discussed under that species, are not present in *C. squamifrons*, namely, the first and second elytral intervals costate. *Conotrachelus lobatus* males, instead, have a spatulate, rounded metauncus, resembling a small, thin translucent spatula (Fig. 3e). *Conotrachelus scoparius* males appear to lack a process at the apex of the hind tibia and instead possess a pair of prominent, testaceus setal tufts (Fig. 14d). The scutellum is elongate narrow in *C. lobatus*, rounded in *C. squamifrons*. In addition, the pronotal punctation in *C. squamifrons*, male and female, is more pronounced, with the diameter of each individual puncture greater than the space between them. The postmedian elytral setal band may be less prominent in *C. squamifrons* than in *C. posticatus*, *C. lobatus* and *C. scoparius*. Despite the name, "squamifrons", other species in this complex bear similar squamosity. The scales in *C. squamifrons* may be more conspicuous because of their strongly contrasting colour in the type series, but this is not a very reliable character to distinguish the species.

#### Distribution

Guatemala, Panama

#### Taxon discussion

In addition to the general remarks above on ways to differentiate males from females, males of *C. squamifrons* can be distinguished from females by interstice 3 being sharply costate, while in some females, it may be subapically interrupted. Males are generally narrower-bodied than females.

Females in the paralectotype series differ from each other in the presence of a subbasal interruption of striae 3 and greater pronotal lateral expansion, resulting in a cordate pronotum e.g. in specimen number NHMUK010801264.

## Analysis

### Results

The specimens intercepted most closely resemble *Conotrachelus posticatus* Boheman, 1837, a widespread species in the U.S. with a distribution extending beyond the U.S. border south as far as Panama (O'Brien and Wibmer 1982). Champion (1904) indicates that *C. posticatus* individuals from Central America differ from those in the U.S. "in having ventral segments less closely punctured." The differences between *C. posticatus* and the recently intercepted *Conotrachelus* adults intercepted on avocado are greater than the intraspecific differences indicated by Champion to be present in *C. posticatus*. Schoof (1942) considered *C. carinifer* Casey, 1892, *C. integer* Casey, 1892 and *C. naso* LeConte, 1876 to be closely related to *C. posticatus.* Upon closer examination of the male genitalia, key characters of the legs of the males (and to a lesser extent, legs of the females), elytral characters, gestalt and other diagnostic features, it became clear that the specimens intercepted included a species that was not *C. posticatus* or any other known U.S. species.

The identity of the male *Conotrachelus*, treated in this study, can be unequivocally determined to be *Conotrachelus lobatus* Champion (Figs 3, 4, 5, 7b). The majority of the females intercepted, with the exception of USNMENT01173425 and USNMENT01448159 (both *C. lobatus*), present a greater challenge and are tentatively identified as members of the *Conotrachelus posticatus* species complex. Three female specimens possessing deep, relatively large pronotal punctures, as in *C. carinifer*, with a reduced median carina, amongst other characters, remain in doubt. A fourth female exhibits features shared by both *C. posticatus* and *C. lobatus*.

## Discussion

Establishing the correct identity of organisms, intercepted at border protection points, has direct implications for food security. Political boundaries do not reflect biological realities and species may or may not occur on both sides of the border. The accidental introduction of a non-native species has the potential to destabilise the delicate balance of natural and agricultural systems. The identification of this weevil, *Conotrachelus lobatus*, putatively associated (loosely or not) with avocado as non-native is significant, since this species closely resembles and may be easily confused with a U.S. native species, *Conotrachelus posticatus*. The risks posed by the non-native weevil *C. lobatus* to U.S. agriculture, particularly commercial U.S. groves of avocados or other resources, are not yet understood, given the limited knowledge we have of the life history of this species. However, some inferences may be made based on historical data.

The identity of *C. lobatus* was confirmed through collaboration between scientists from two museums with extensive and historical research collections, the Natural History Museum in London and the U.S. Museum of Natural History in Washington, DC. The linking of the newly intercepted species to an older, already described taxon provides valuable baseline data, especially as it pertains to the original native range of the species, or at least its area of occurrence more than a century ago. This type of historical information is often poorly known or unknown for invasive/alien organisms, but is crucial for prospecting for natural enemies for possible implementation of biological control programmes (e.g. Orlova-Bienkowskaja and Volkovitsh 2017) or predicting additional host plants of an emerging pest species. Such opportunities could not exist for a species described as new from an adventive population.

In addition to the 120 year-old specimens described by Champion, two addtional series of *Conotrachelus lobatus* collected in the 1970s and 80s are confirmed. One is a series of specimens in the USNM weevil collection, identical to the intercepted specimens, which were tentatively identified by Donald Whitehead (USDA, SEL, deceased) as *C. lobatus*. Whitehead (1979) cited a number of undetermined species collected in Central Mexico as part of an avocado pest survey, which he did not consider to be avocado pests because they did not belong to "group r'", the avocado pest group, in the key by Champion (1904). These specimens in the USNM collection may correspond to some of the ones cited by Whitehead. Labels on these specimens read: "Mex. Mich. Uruapan Avocado Pest Survey VII.73" // "Jarred from tree. Burgess Brownsville, TX". These specimens suggest the presence of *C. lobatus* in the avocado groves of Michoacán, Western Mexico, as far back as 1973. The label data is confusing since one label cites Mexico, Michoacan and the other label indicates Brownsville TX. We interpret the first label to indicate the origin of the specimens and the second label as the inspection port, which, in this instance, is in Brownsville, TX. Burgess is interpreted to be the name of the inspector or surveyor. The interpretation of these labels is based on a similar practice used by the senior author when transcribing locality/collection data from interception forms that accompany specimens sent to her for identification.

The second is a series of specimens in the Charles W. O'Brien collection (CWOB). O'Brien confirmed the identity of these specimens based on comparison with images sent to him of the type, which are the same images included in this manuscript. O'Brien sent the following information when the label data was requested: "One important specimen says 'Uruapan Mich [Michoacán, Mexico],/ Junio – 1989/ 1600 M/ Leon Llandera' // 'Hosp. Persea americana'. Some from Nayarit say 'pine/oak', one 'oak' only, which probably refer to habitat.".

*Conotrachelus posticatus* and species in this complex are known to be associated with acorns of *Quercus* spp. (Schoof 1942). Specimens of *Conotrachelus posticatus* at USNM from Michoacán (Jicalan), Mexico were reared from acorns of *Quercus obtusata* Bonpl. *Conotrachelus* species are also routinely collected near light sources (Schoof 1942). In addition, specimens of *C. lobatus* have been intercepted (not necessarily intimately associated) with commodities, other than avocados, such as papaya.

In summary, the following information is known about *Conotrachelus lobatus*:

- collected approximately 120 years ago in west-central Durango state, Mexico;

- collected between 43-27 years ago in/near avocado groves in the west-central Mexican states of Nayarit and Michoacán, the latter being the heart of the avocado growing region of Mexico;

- collected in/near oak stands;

- putative sister species associated with oaks (*Conotrachelus posticatus* complex);

- belongs to a group of weevils that are readily attracted to lights;

- intercepted (possibly incidentally) with at least two different types of commodities: avocados and papaya;

- usually collected/intercepted during the months of June and July.

Given the resemblance between *Conotrachelus lobatus* and several *Conotrachelus* species native to the U.S.A., we have provided numerous distinguishing features to prevent type I and II errors (false positives and false negatives). Sexual dimorphism makes identification of single specimens challenging and one must exercise caution when only a single sex is present, particularly the female.

While Schoof (1942)'s monograph of midwestern U.S. *Conotrachelus* is extremely well produced and useful, the Biologica Centrali-Americana (Champion 1904), although somewhat outdated, is also appropriate to consult in order to identify weevils from south of the U.S. border. A comprehensive study of *Conotrachelus posticatus* and its close relatives, especially with reference to species occurring in the USA, is sorely needed in order to understand the limits of this highly variable species.

## Supplementary Material

XML Treatment for Conotrachelus lobatus

XML Treatment for Conotrachelus posticatus

XML Treatment for Conotrachelus scoparius

XML Treatment for Conotrachelus squamifrons

## Figures and Tables

**Figure 1a.**
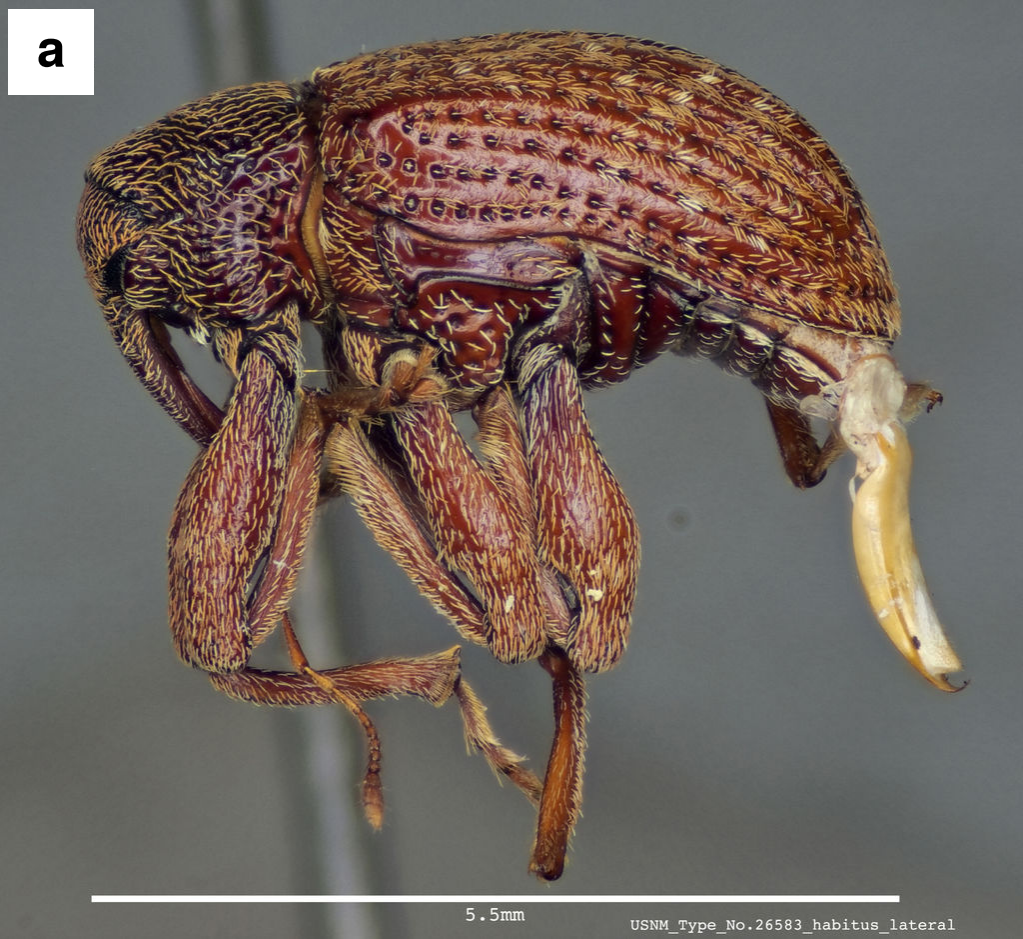
Lateral view.

**Figure 1b.**
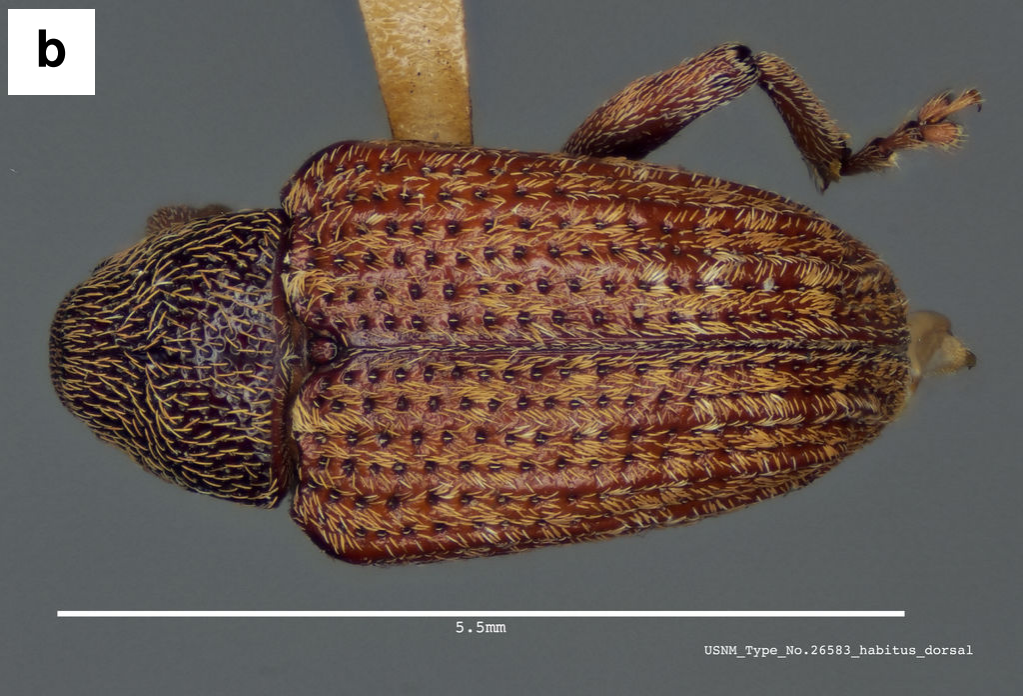
Dorsal view.

**Figure 1c.**
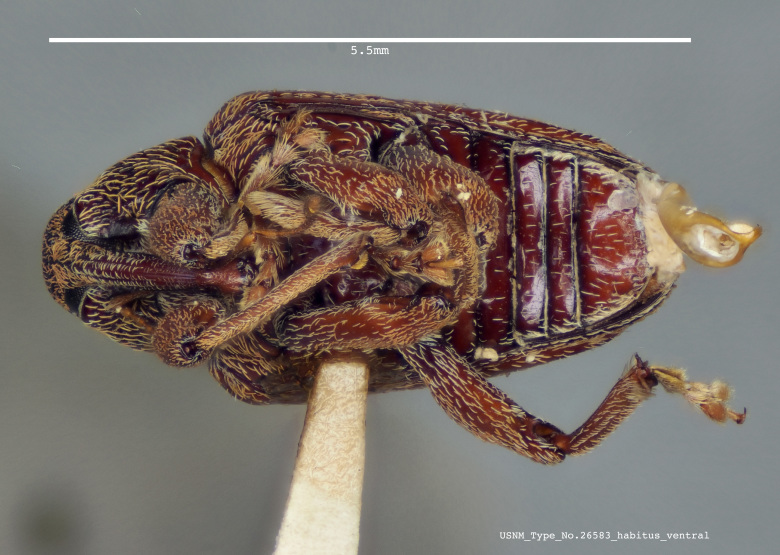
Ventral view.

**Figure 1d.**
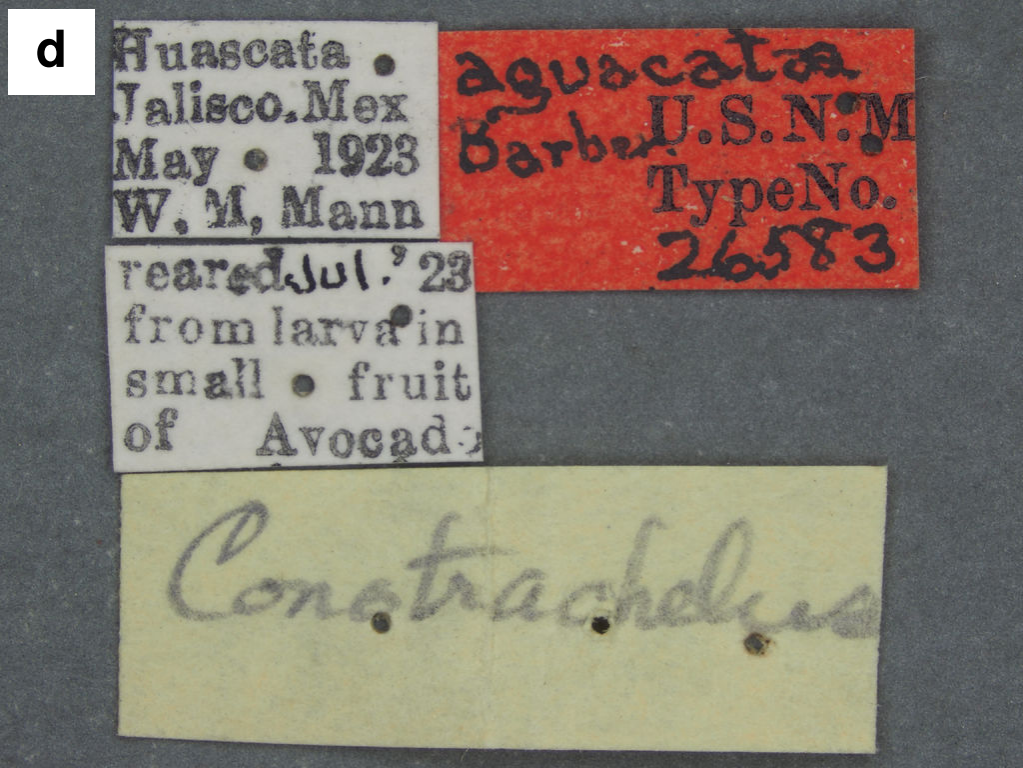
Labels.

**Figure 2a.**
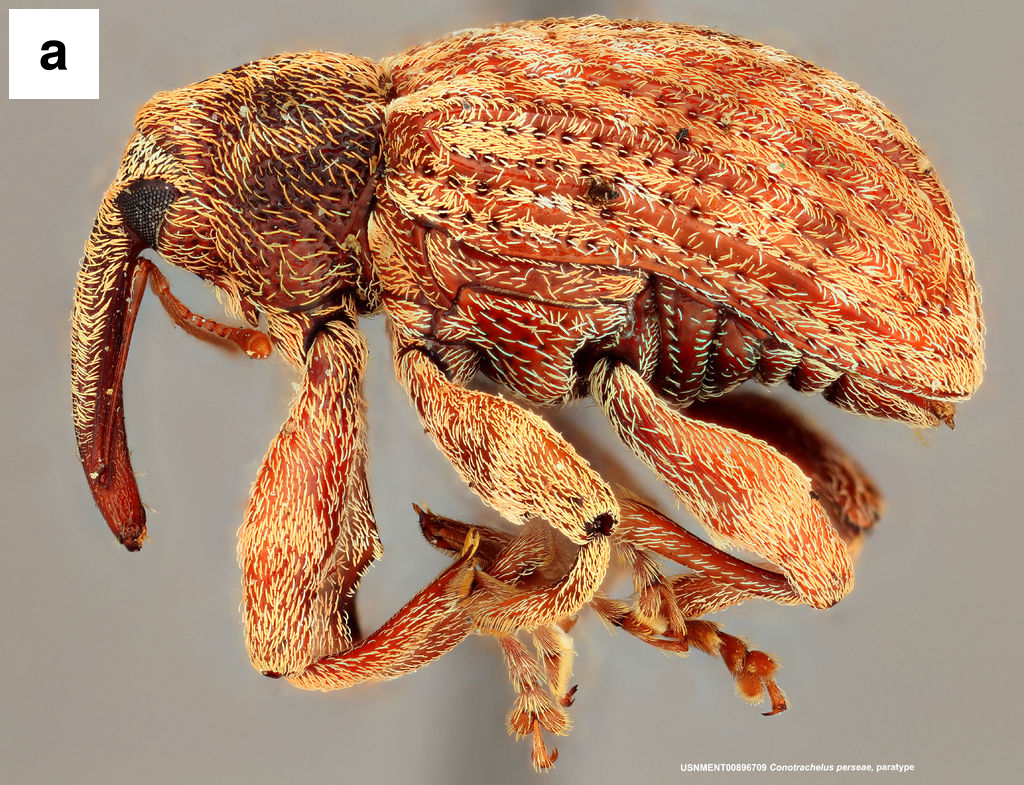
Lateral view.

**Figure 2b.**
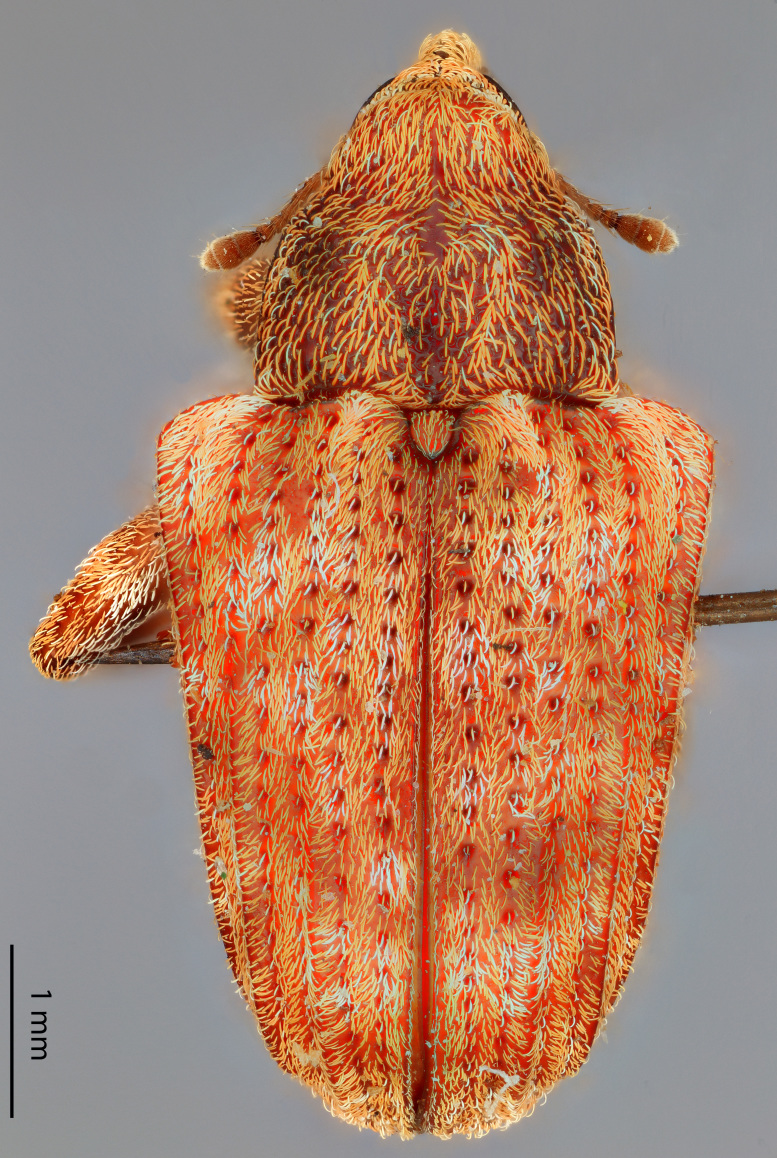
Dorsal view.

**Figure 2c.**
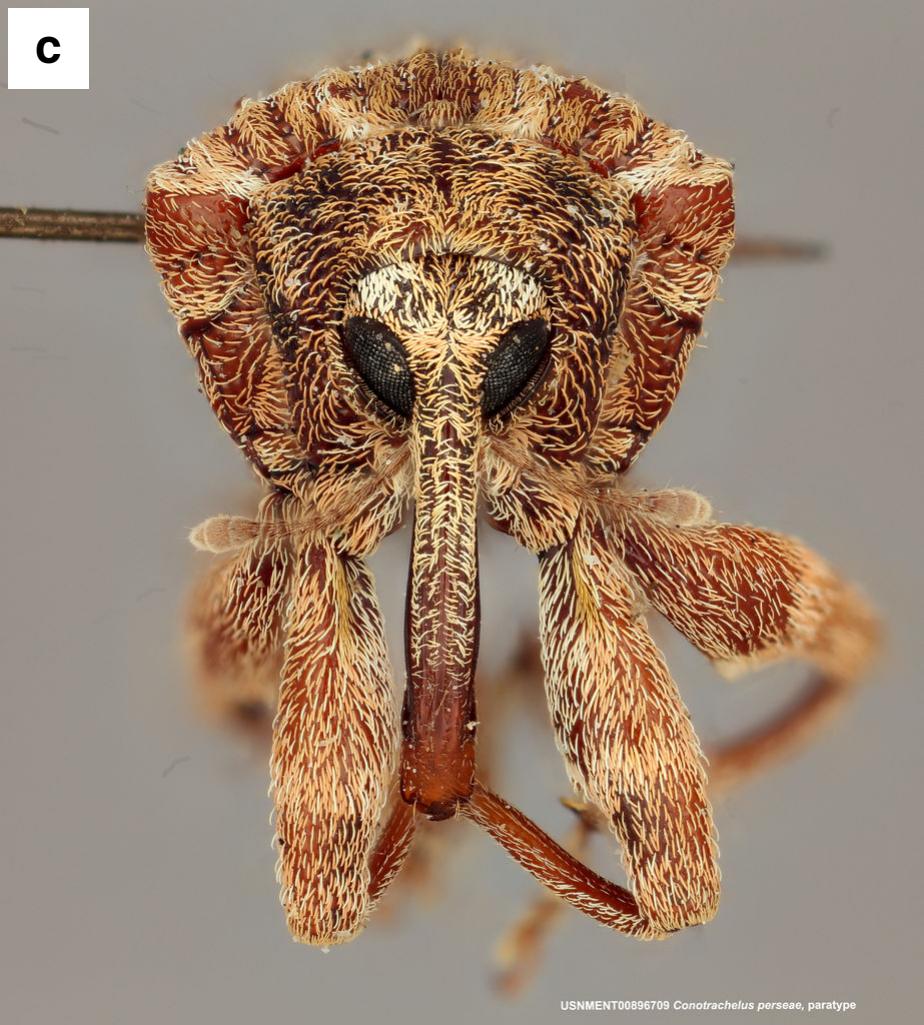
Anterior view.

**Figure 3a.**
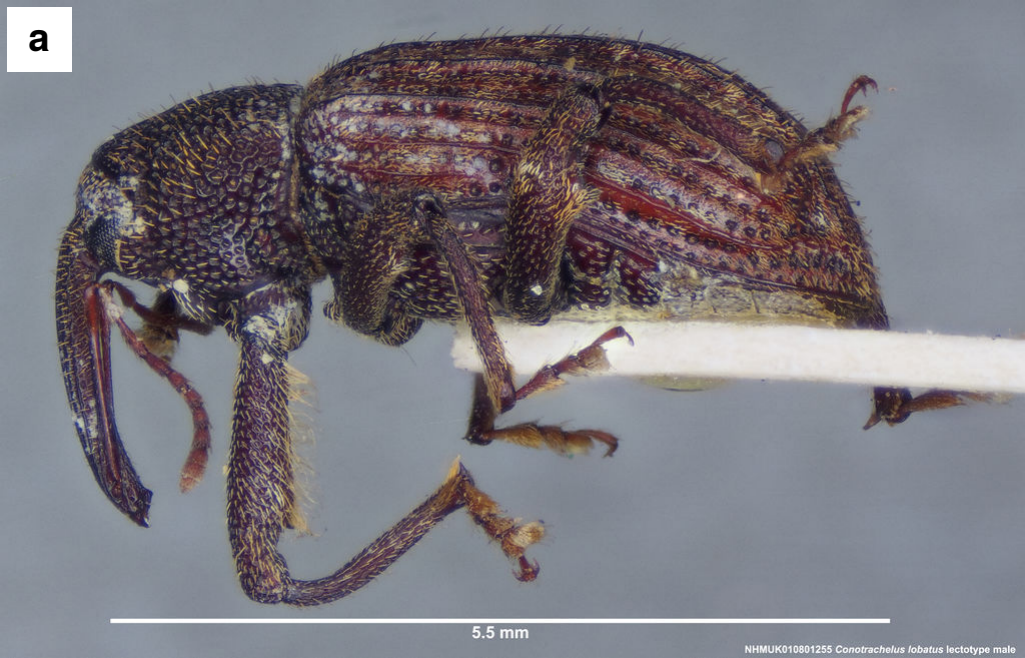
Lateral view.

**Figure 3b.**
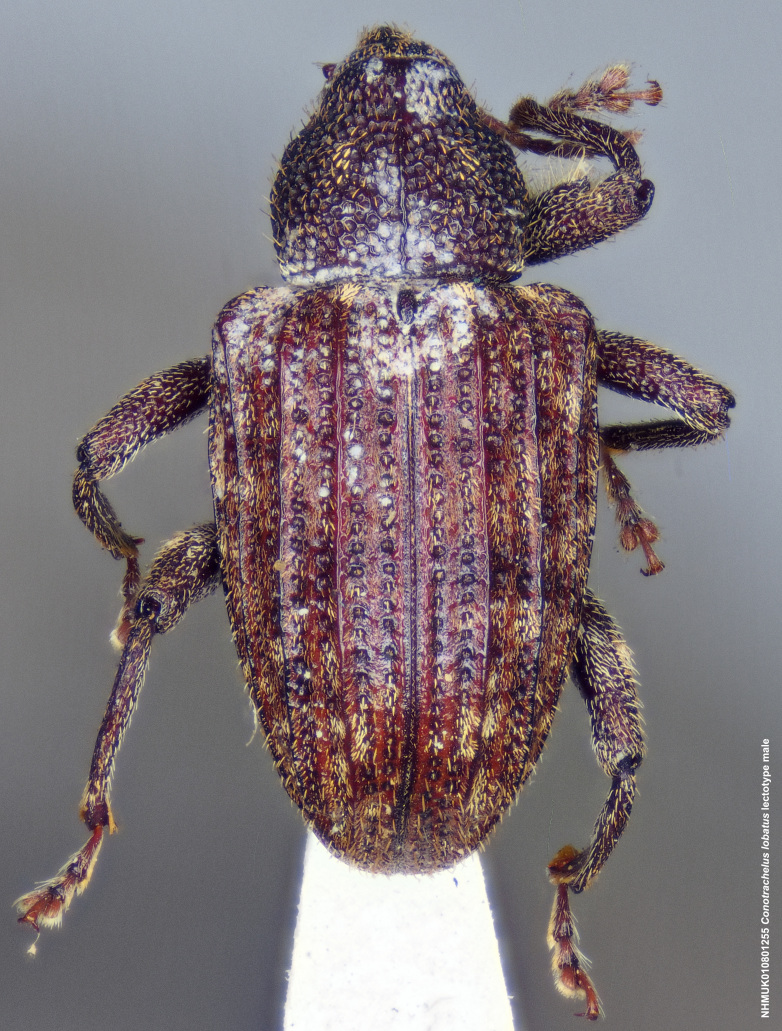
Dorsal view.

**Figure 3c.**
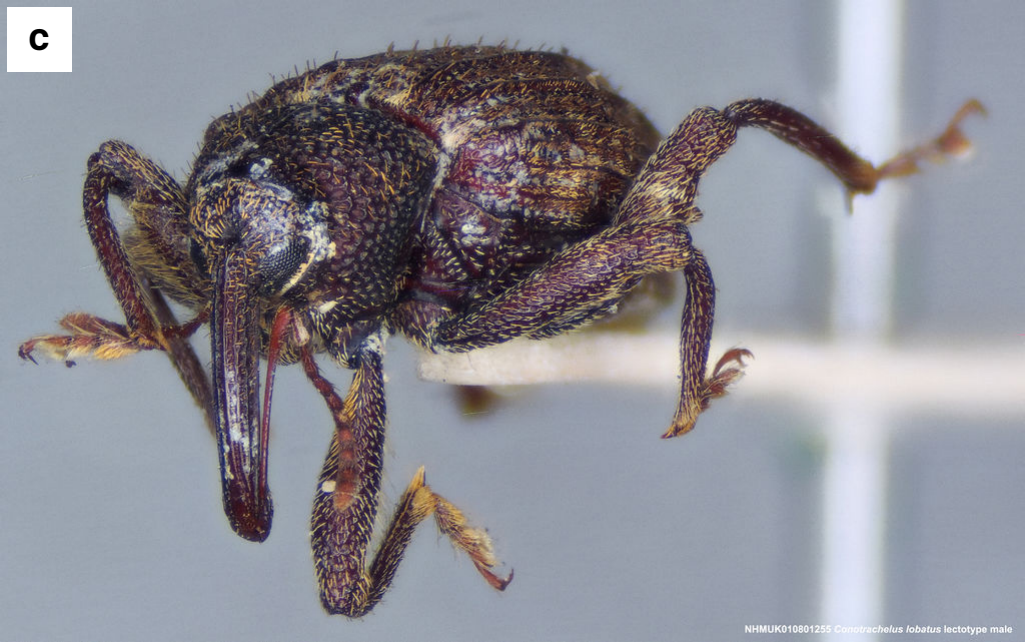
Oblique lateral view.

**Figure 3d.**
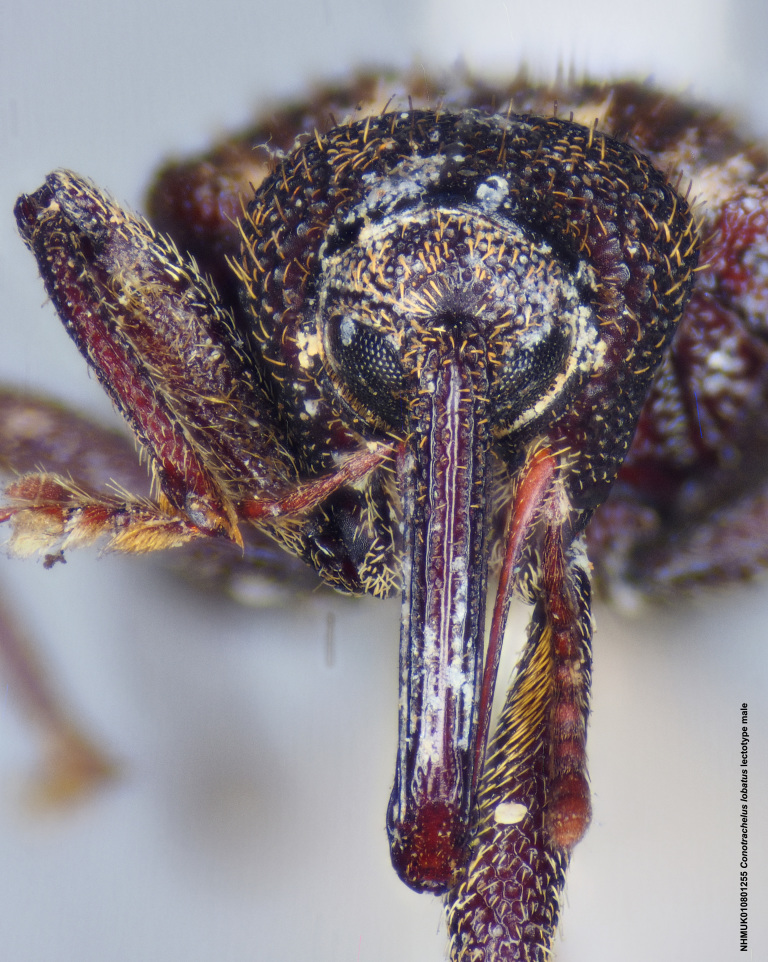
Anterior view.

**Figure 3e.**
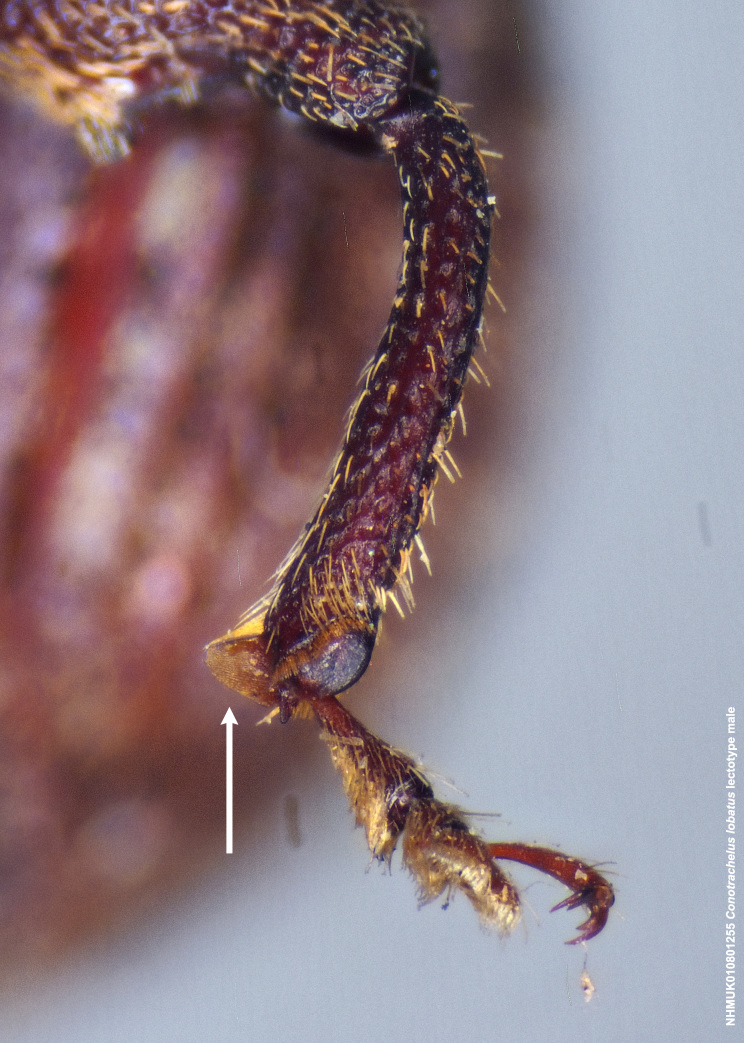
Detail, hind leg, lateral view.

**Figure 3f.**
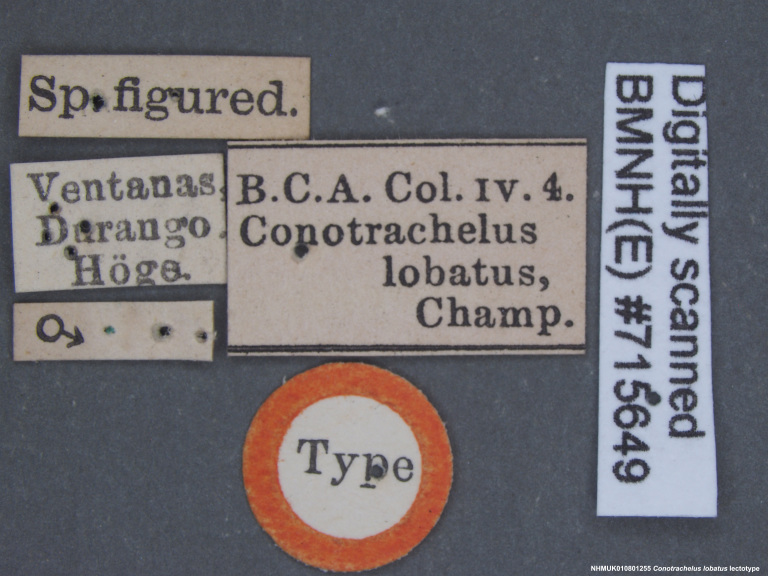
Labels.

**Figure 4a.**
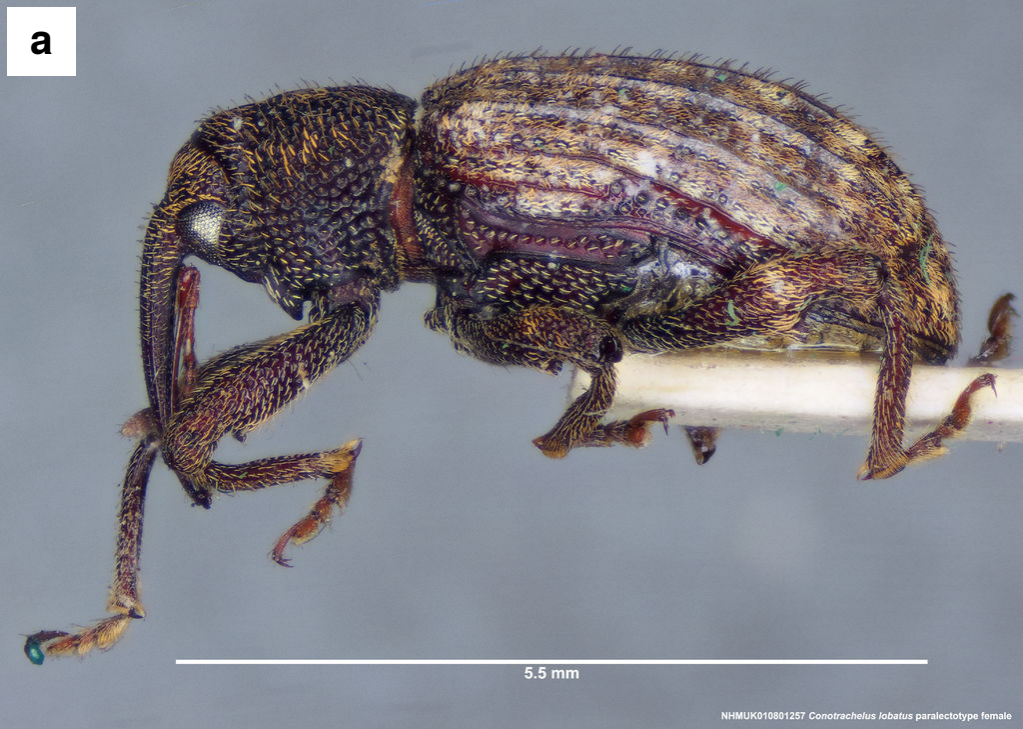
Lateral view.

**Figure 4b.**
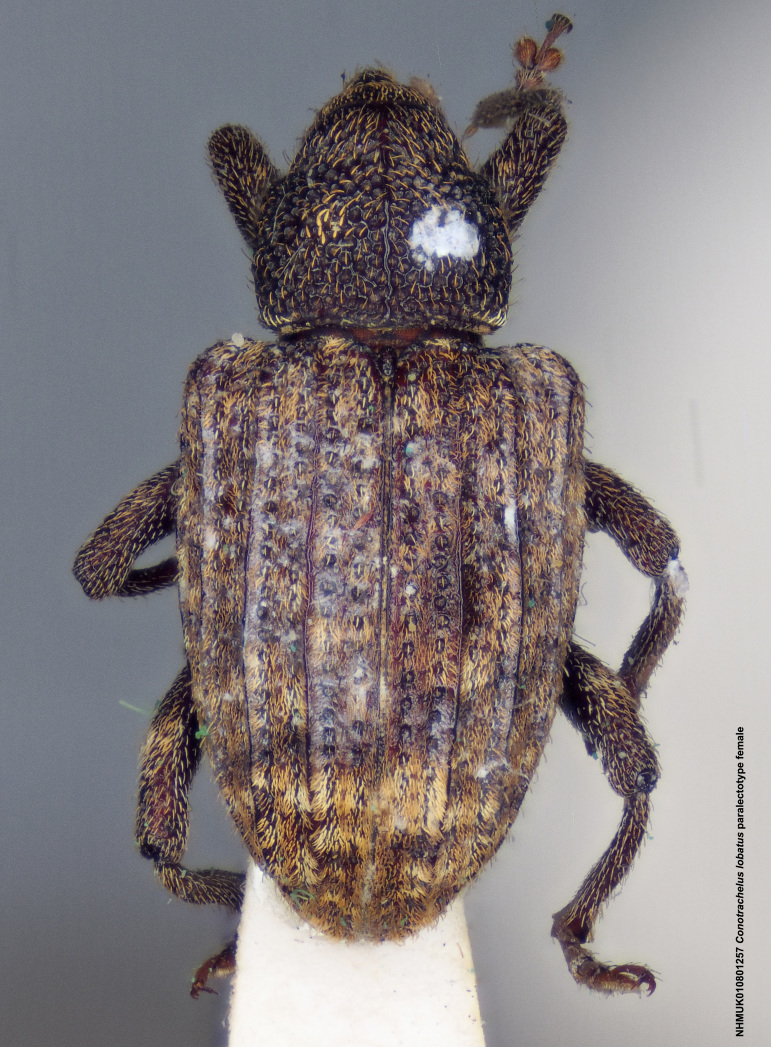
Dorsal view.

**Figure 4c.**
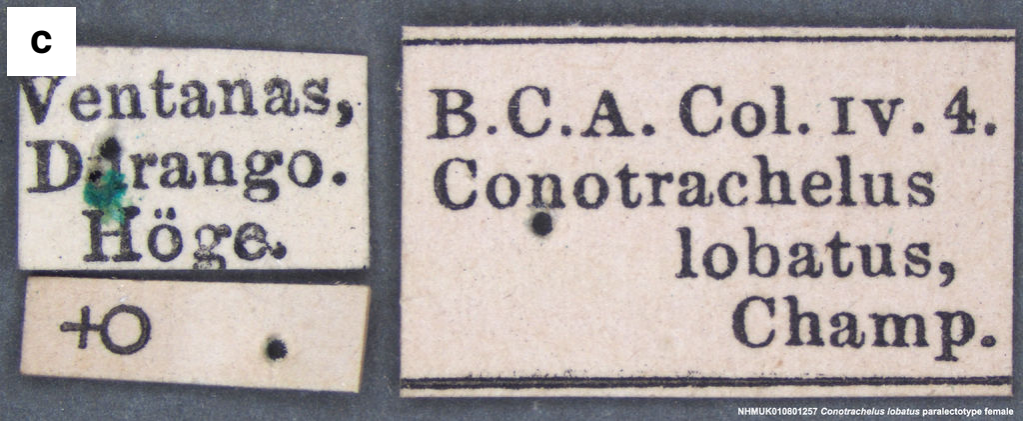
Labels.

**Figure 5a.**
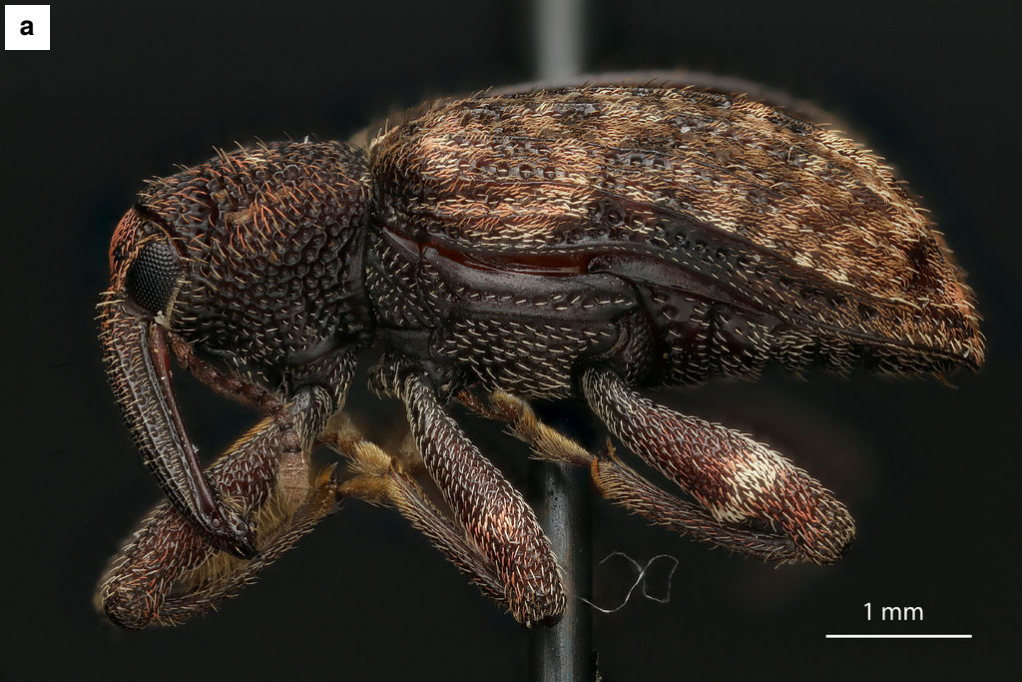
Male.

**Figure 5b.**
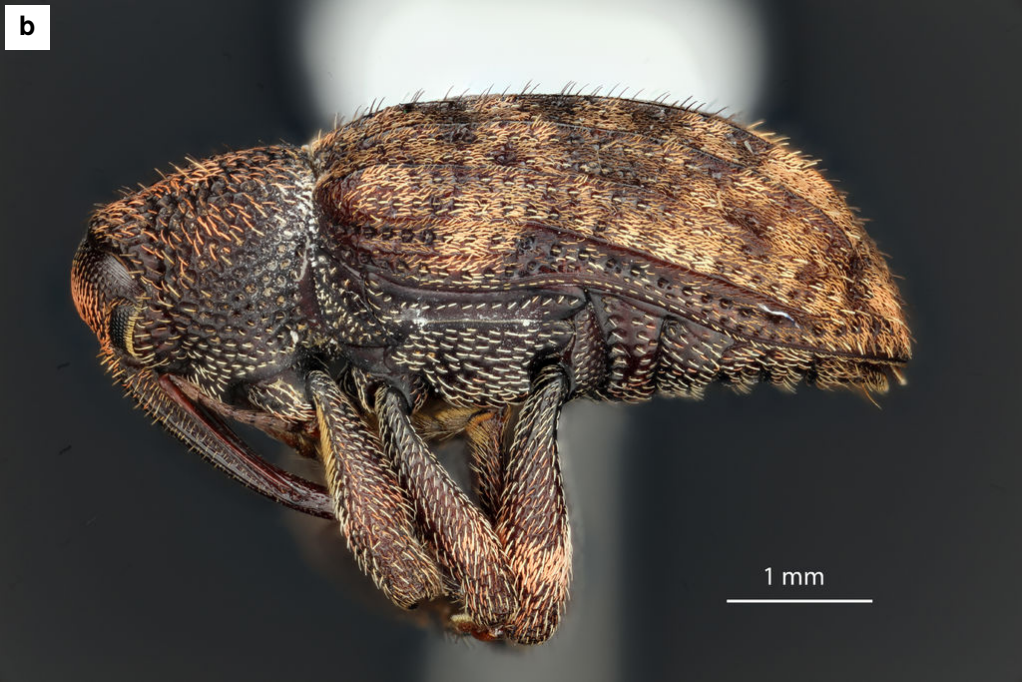
Female (USNMENT01173425).

**Figure 6a.**
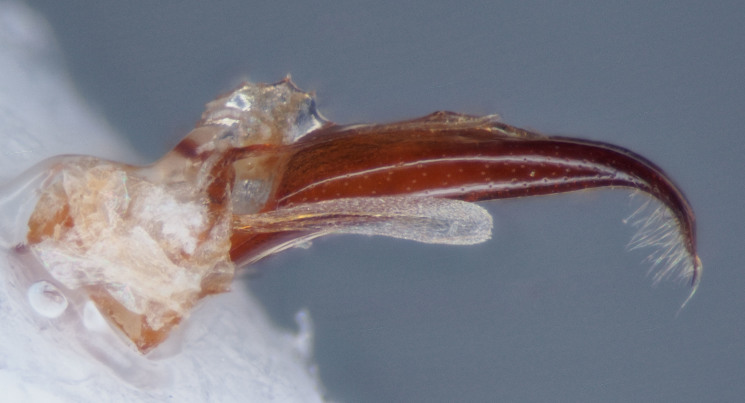
Lateral view.

**Figure 6b.**
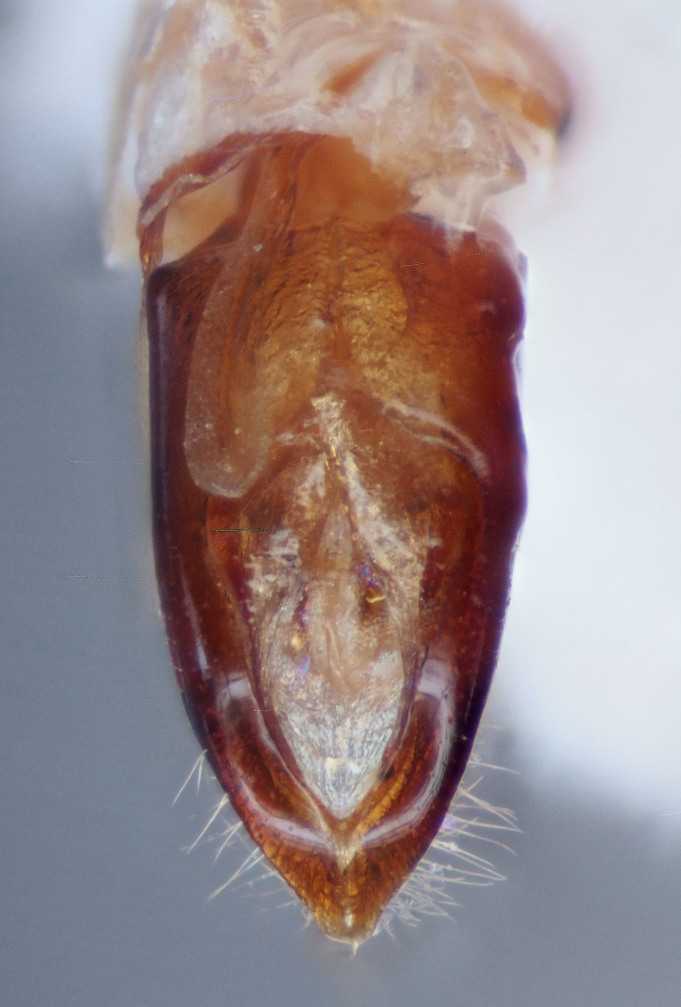
Dorsal view.

**Figure 6c.**
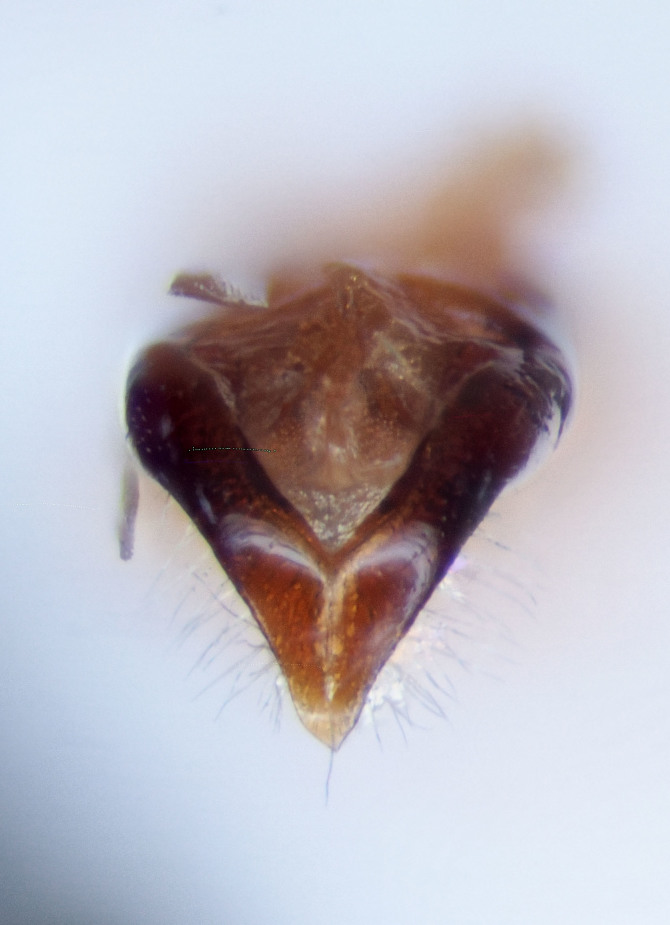
Anterior view.

**Figure 7a.**
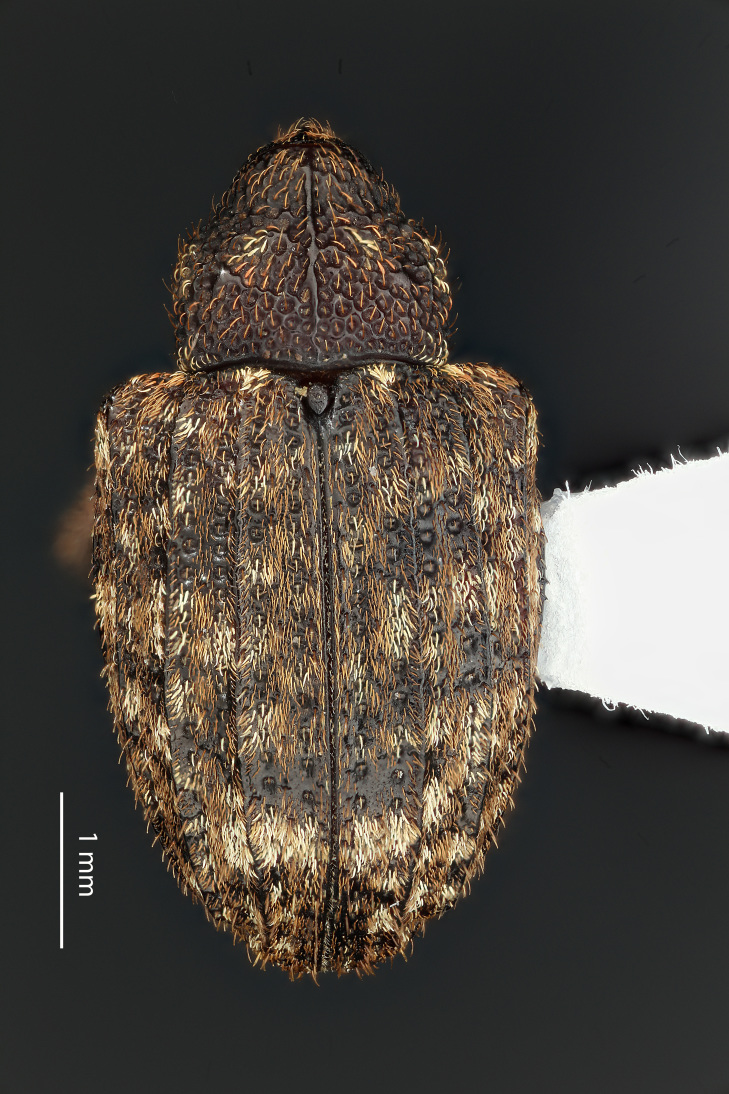
Conotrachelus nr posticatus, female (USNMENT01173426).

**Figure 7b.**
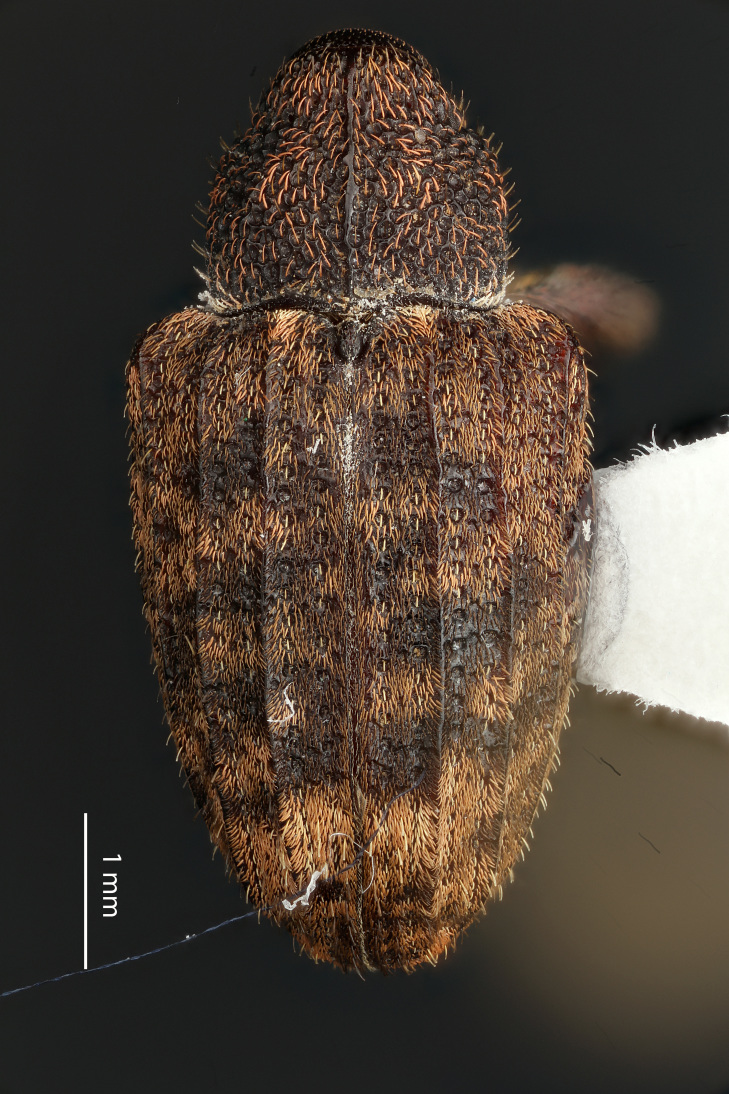
*Conotrachelus lobatus*, female (USNMENT01173425).

**Figure 9a.**
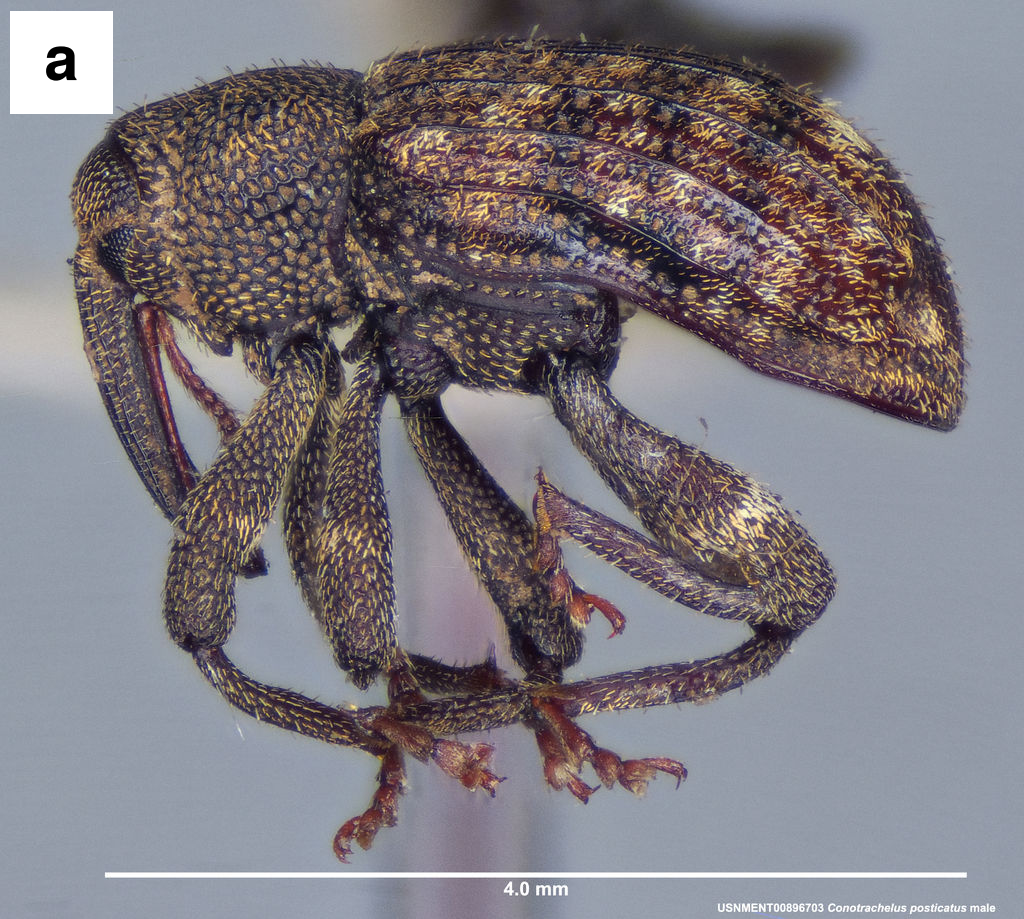
Lateral view.

**Figure 9b.**
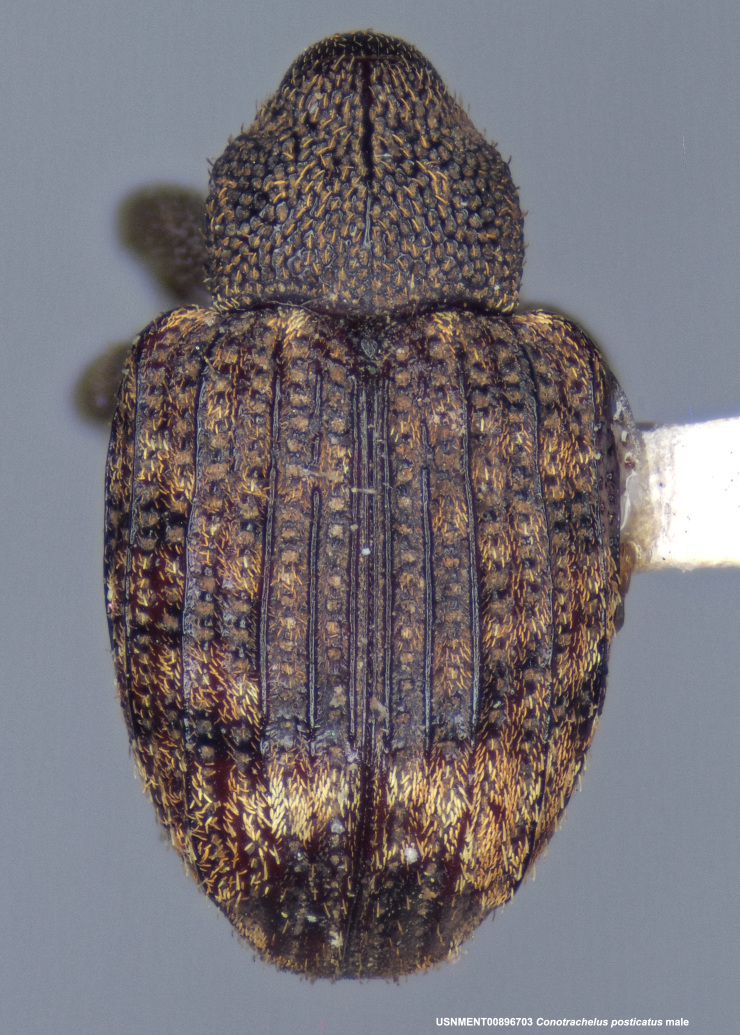
Dorsal view.

**Figure 9c.**
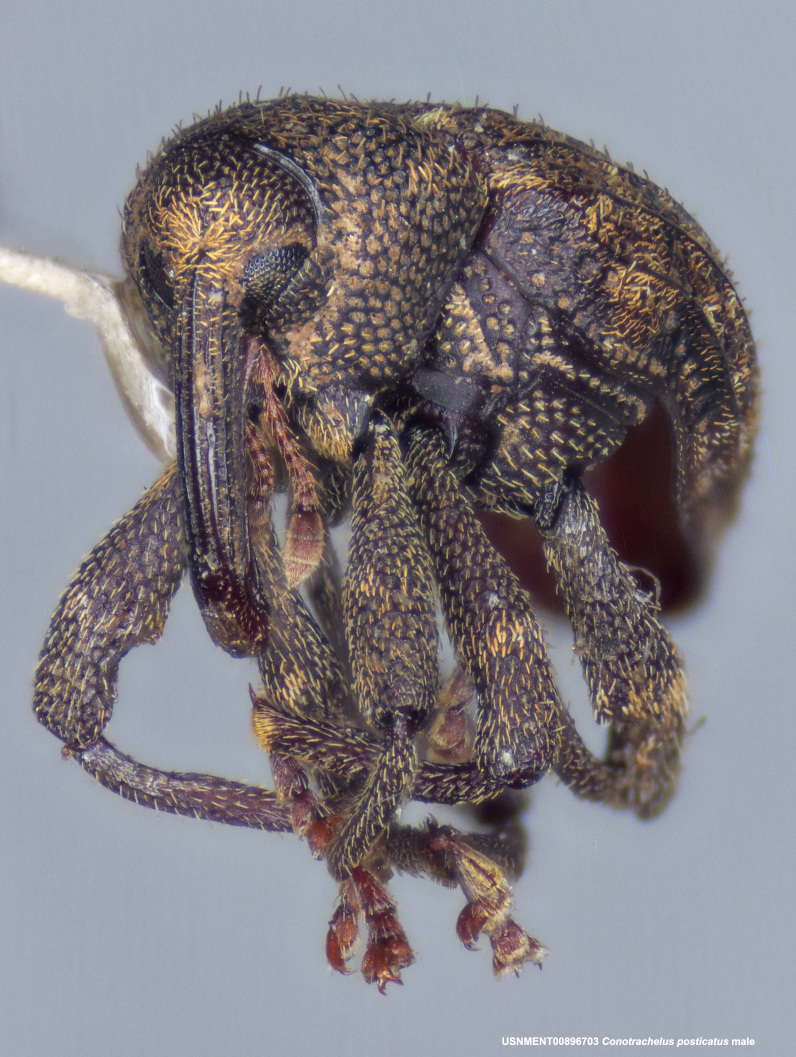
Oblique-lateral view.

**Figure 9d.**
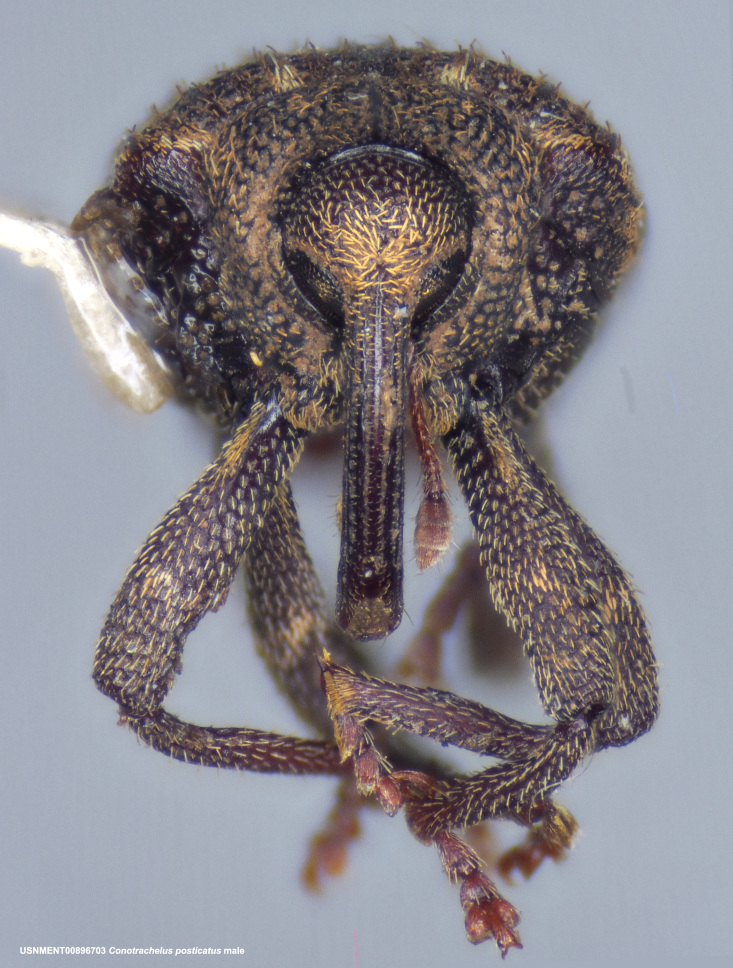
Anterior view.

**Figure 9e.**
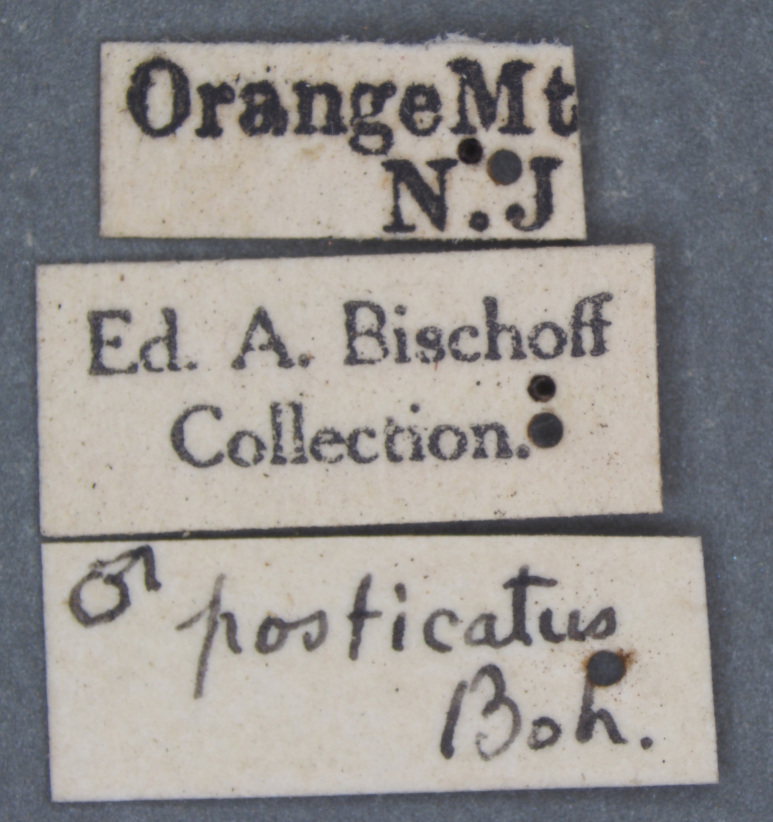
Labels.

**Figure 10a.**
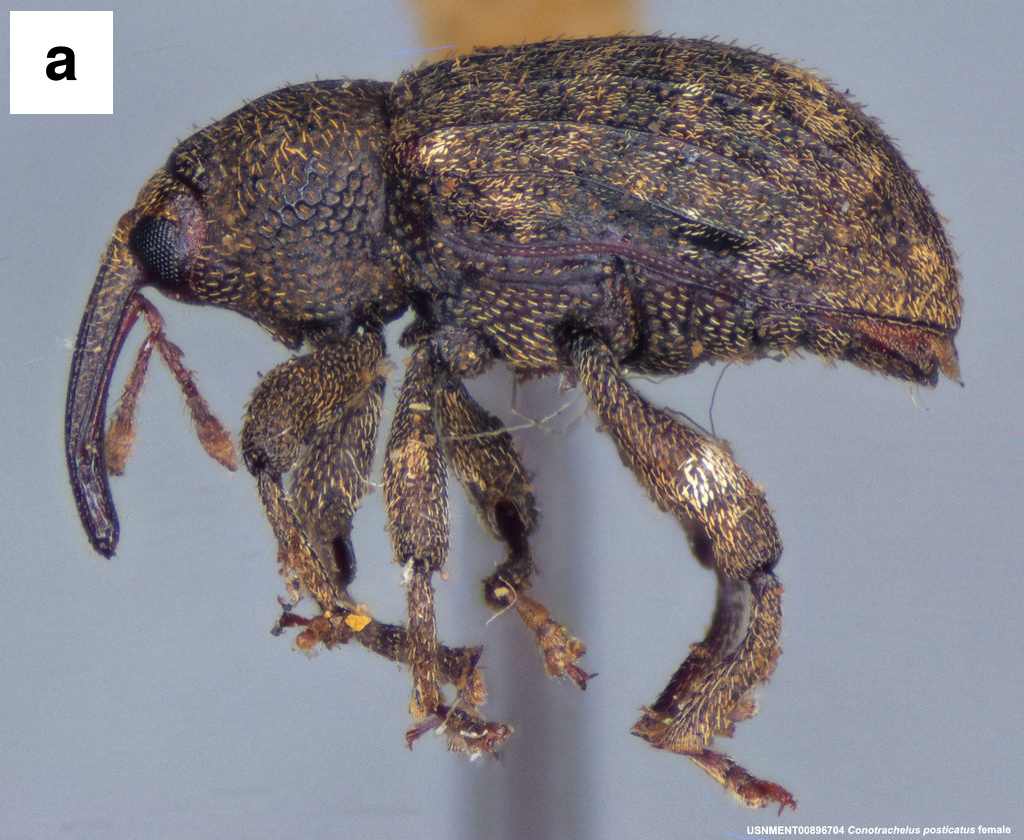
Lateral view.

**Figure 10b.**
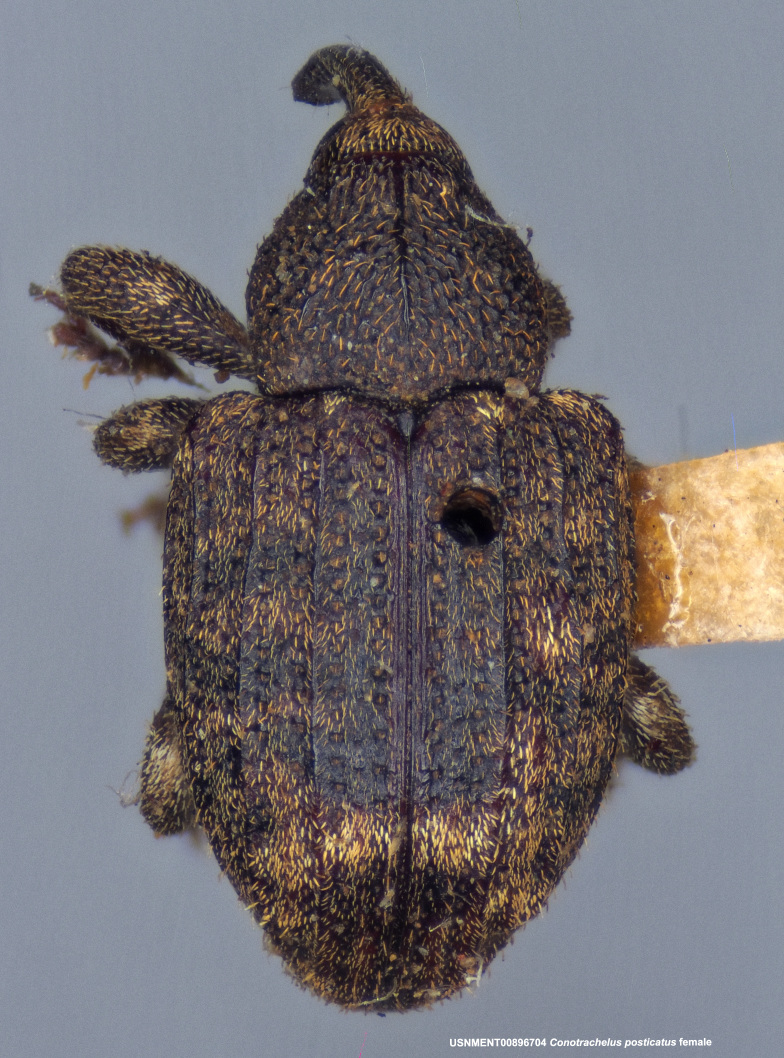
Dorsal view.

**Figure 10c.**
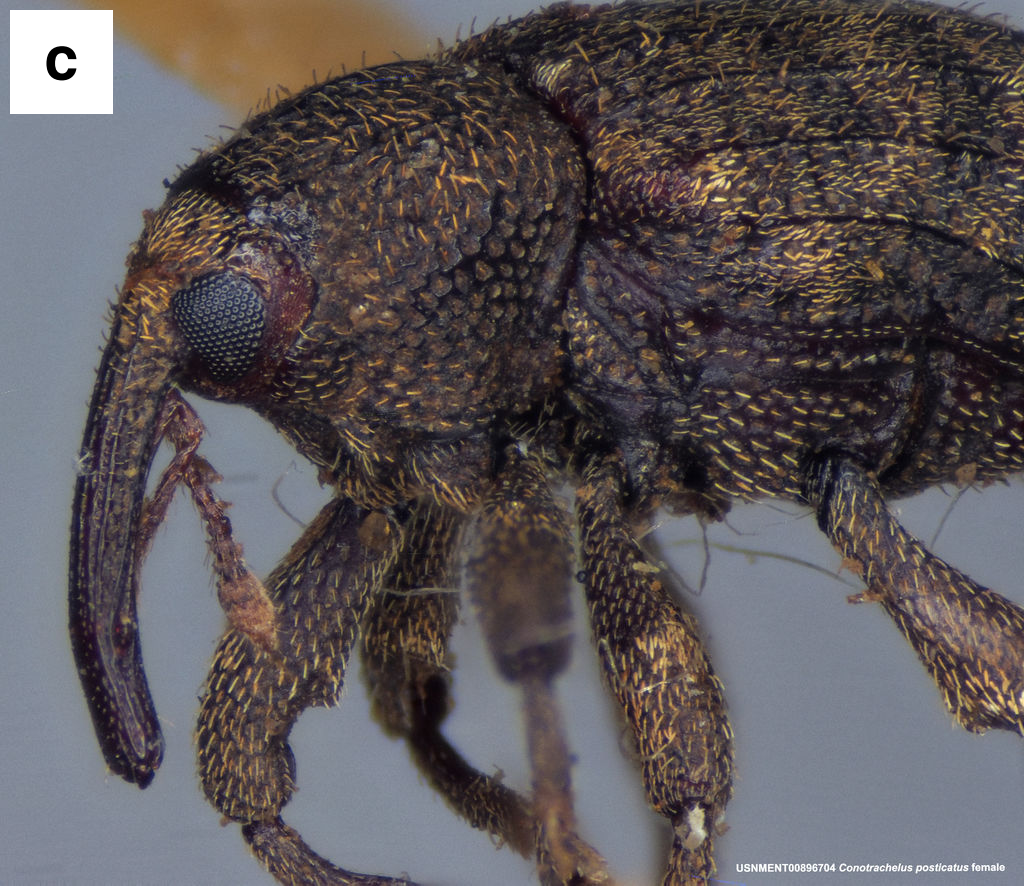
Oblique-lateral view.

**Figure 10d.**
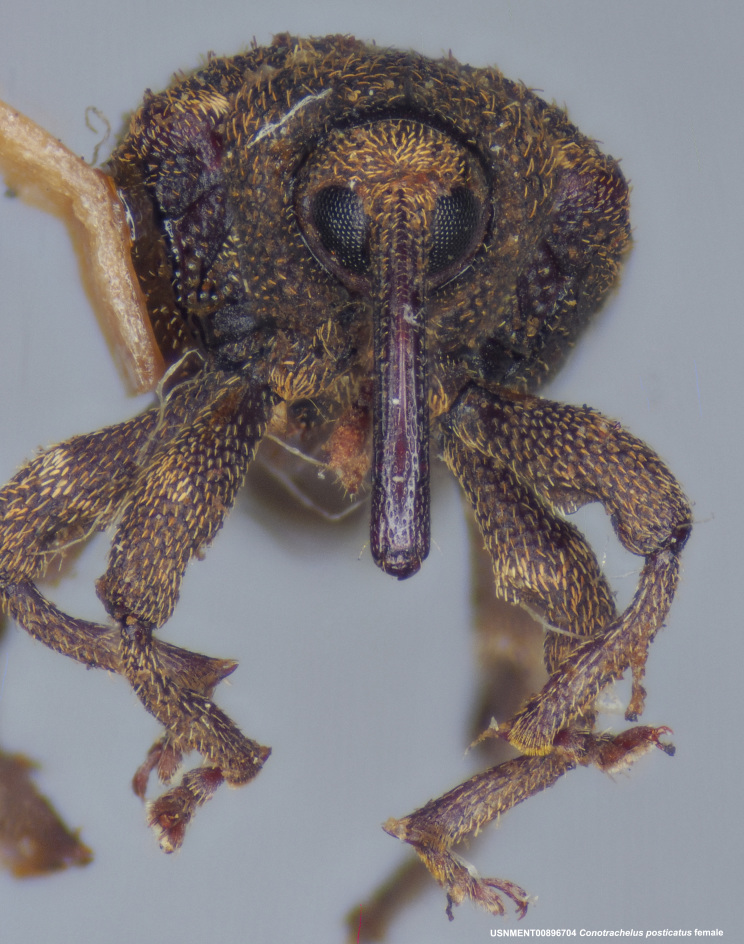
Anterior view.

**Figure 10e.**
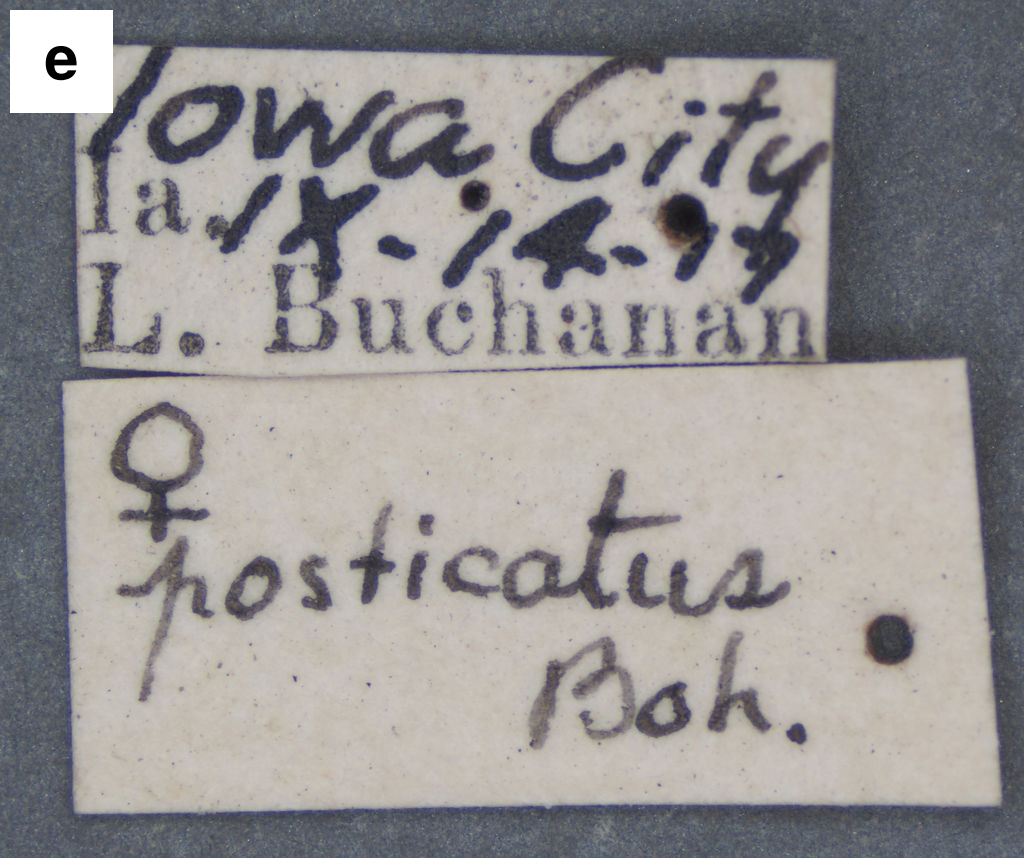
Labels.

**Figure 11a.**
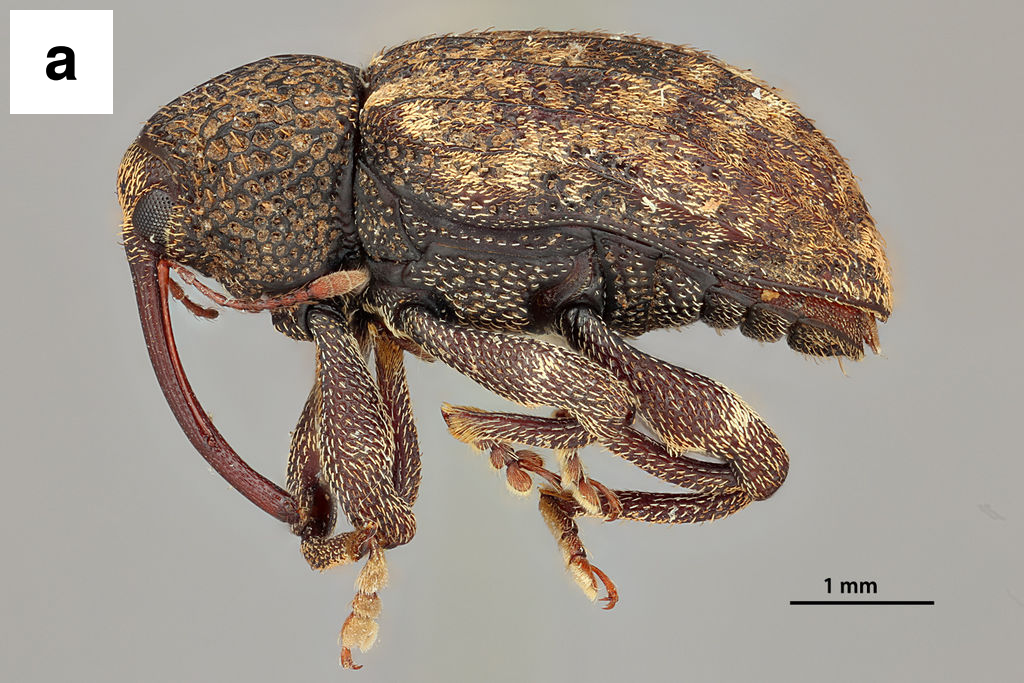
Lateral view

**Figure 11b.**
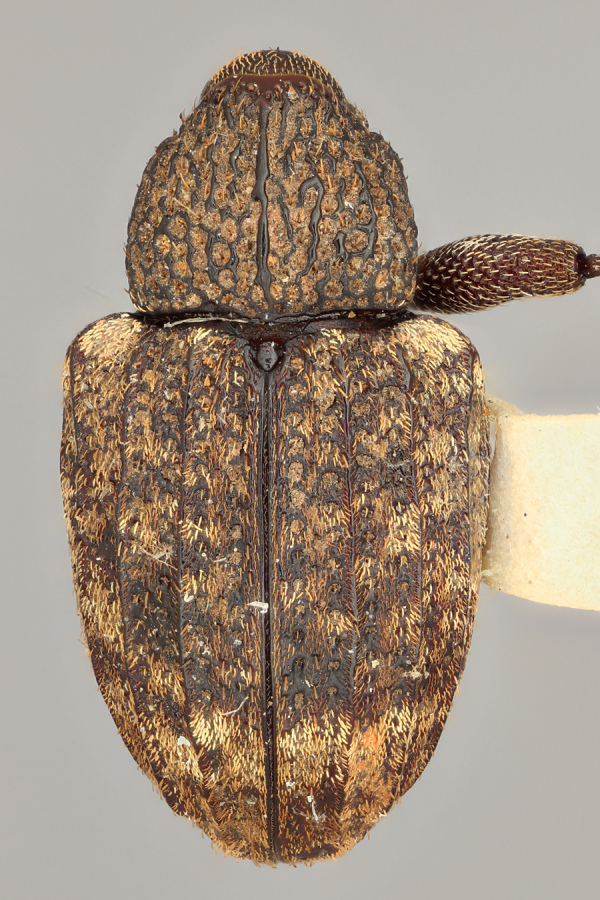
Dorsal view

**Figure 11c.**
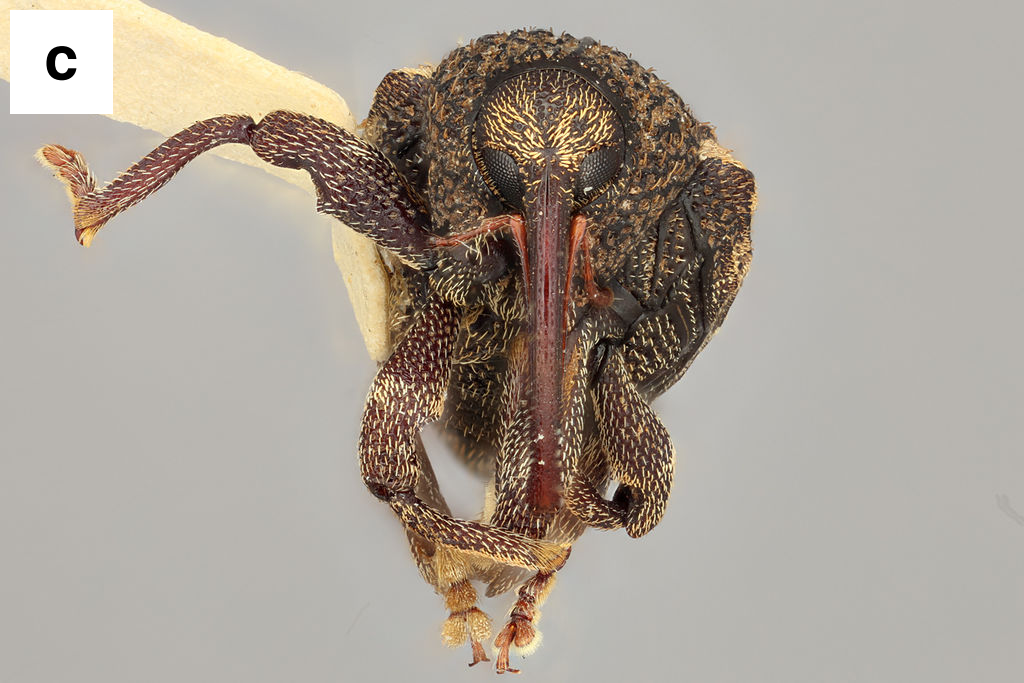
Anterior view

**Figure 11d.**
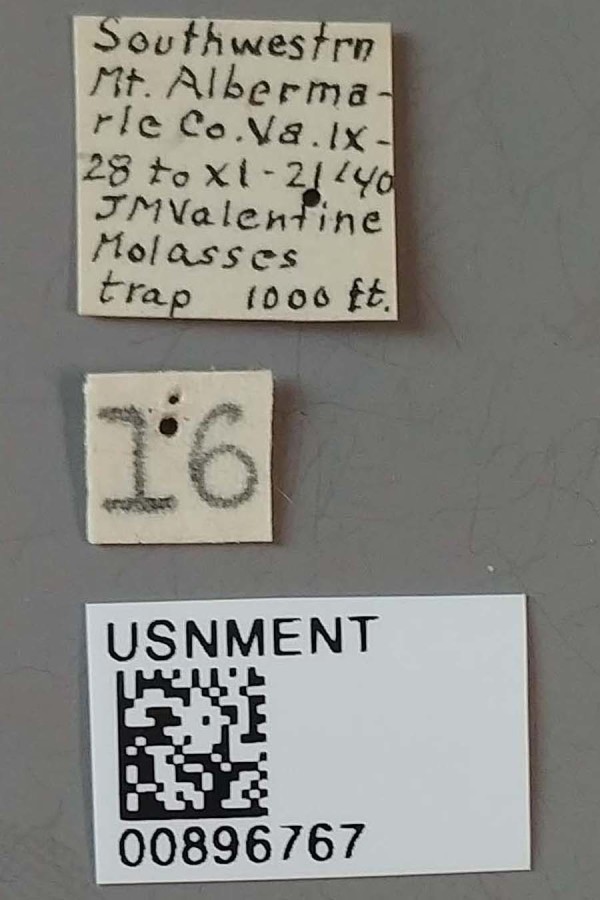
Labels

**Figure 12a.**
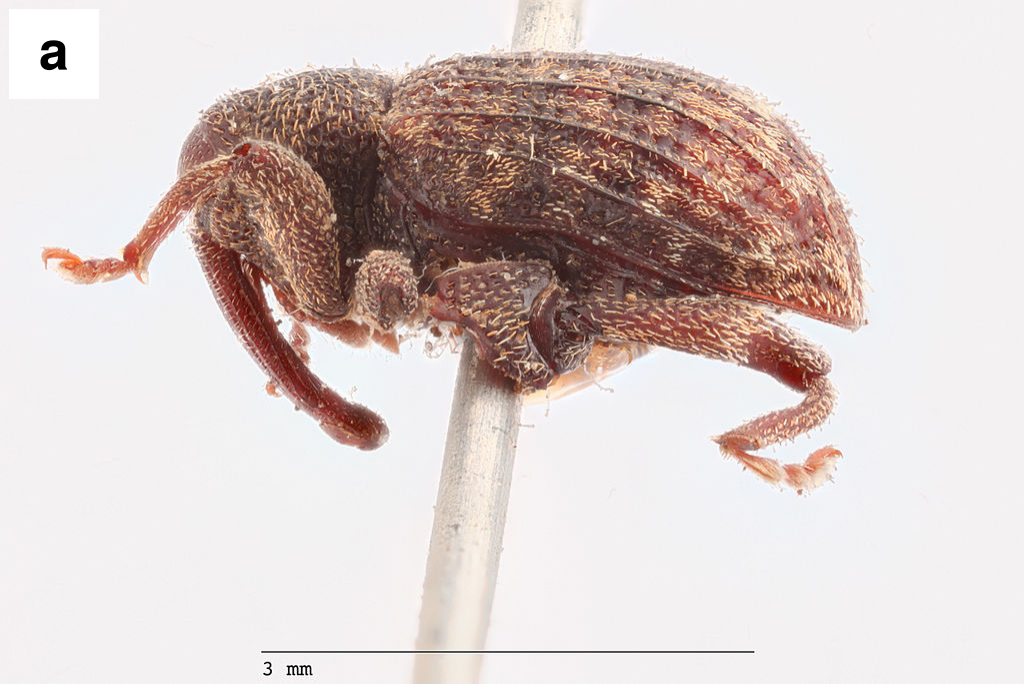
Lateral view.

**Figure 12b.**
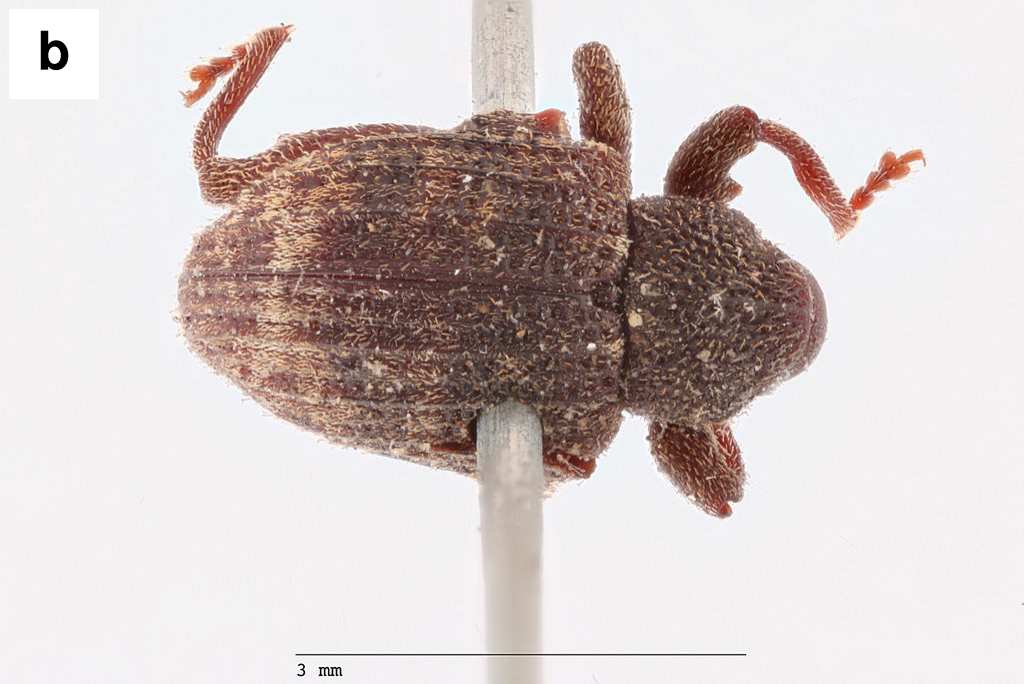
Dorsal view.

**Figure 12c.**
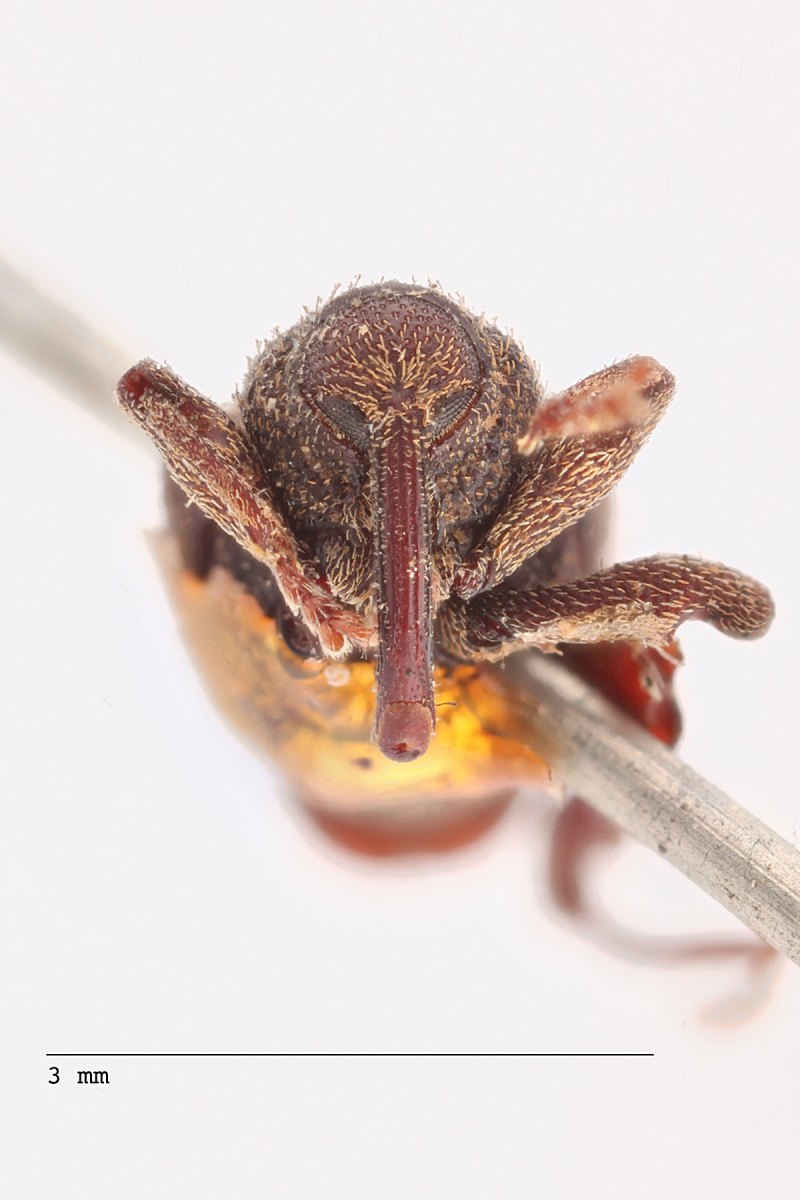
Anterior view.

**Figure 12d.**
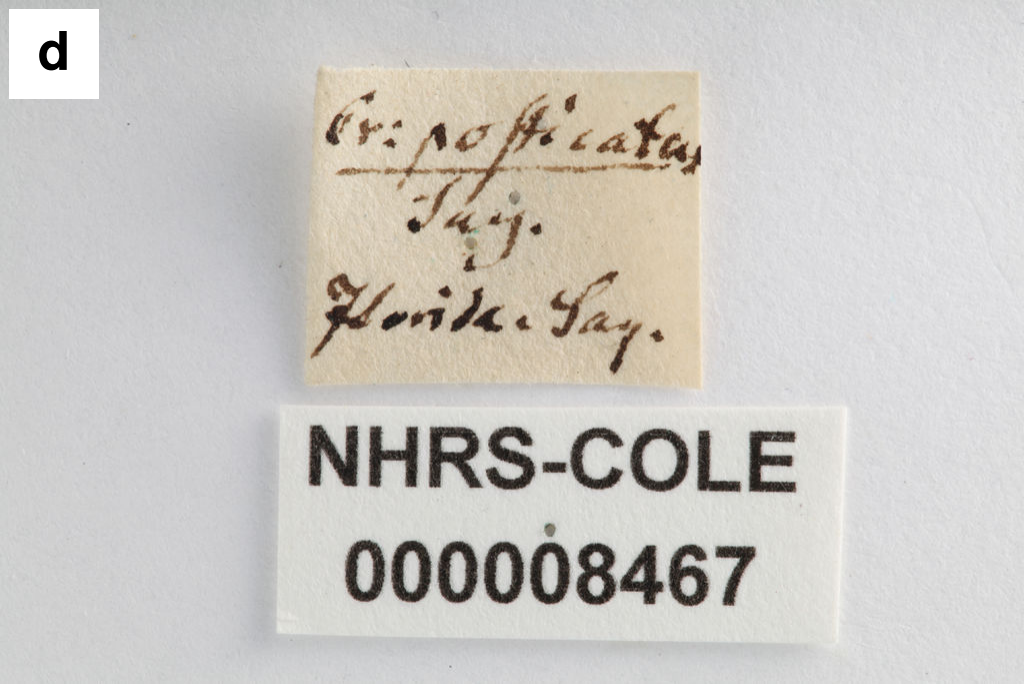
Specimen labels.

**Figure 12e.**
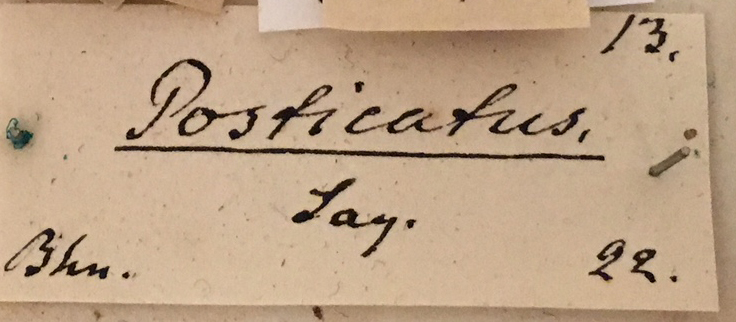
Drawer label in Schoenherr collection.

**Figure 13a.**
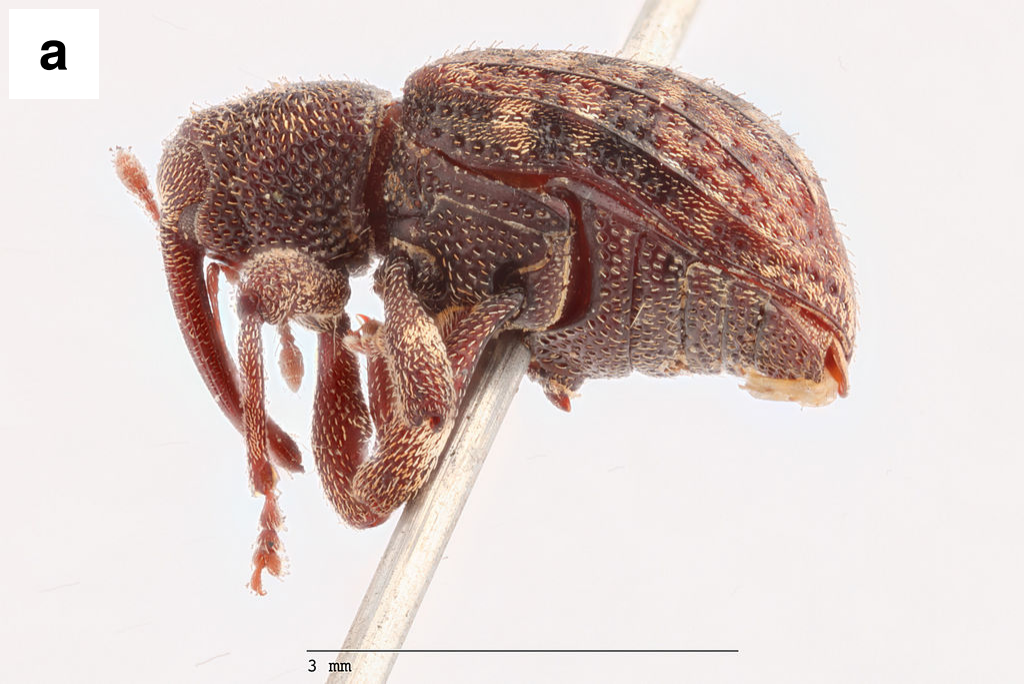
Lateral view.

**Figure 13b.**
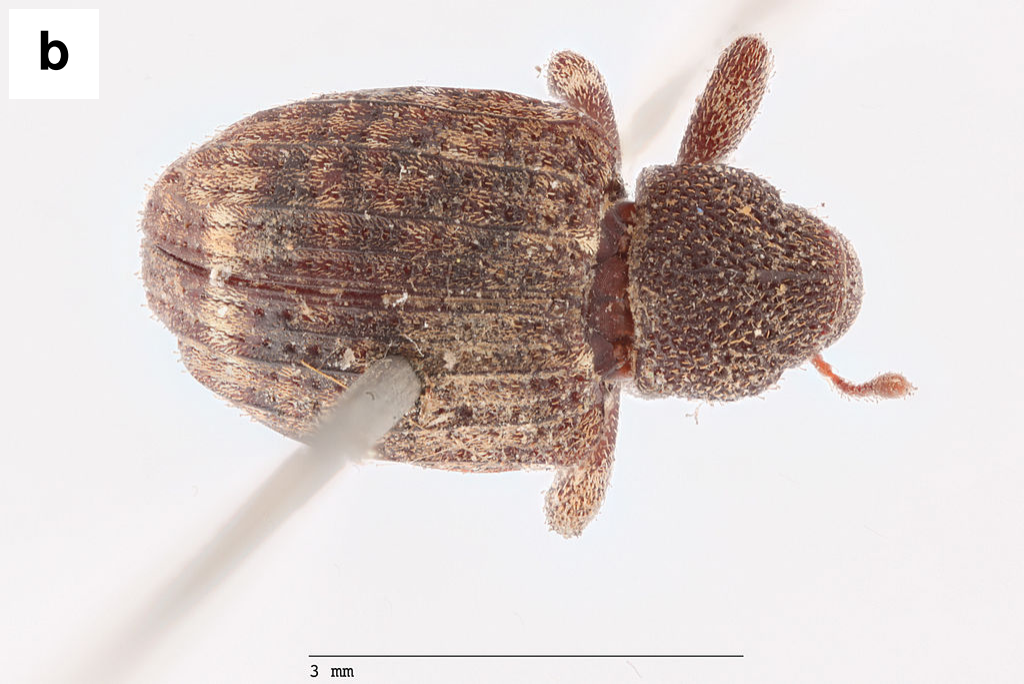
Dorsal view.

**Figure 13c.**
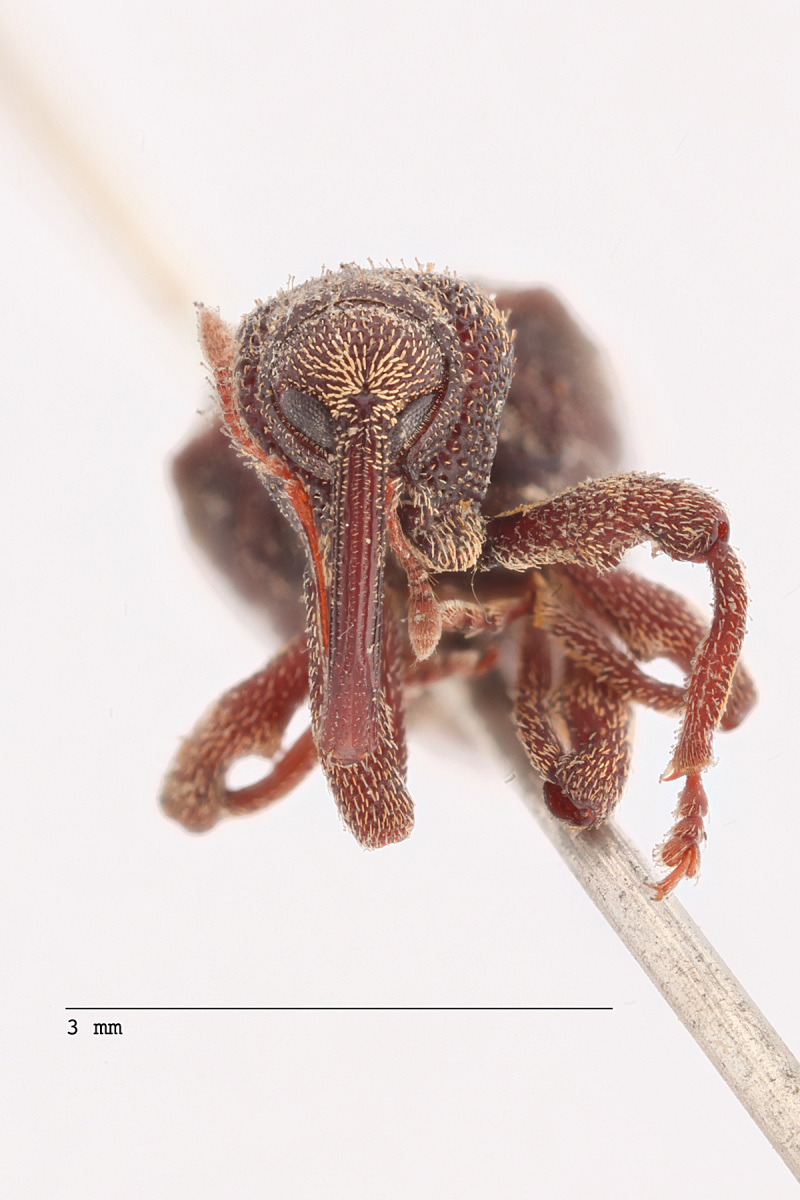
Anterior view.

**Figure 13d.**
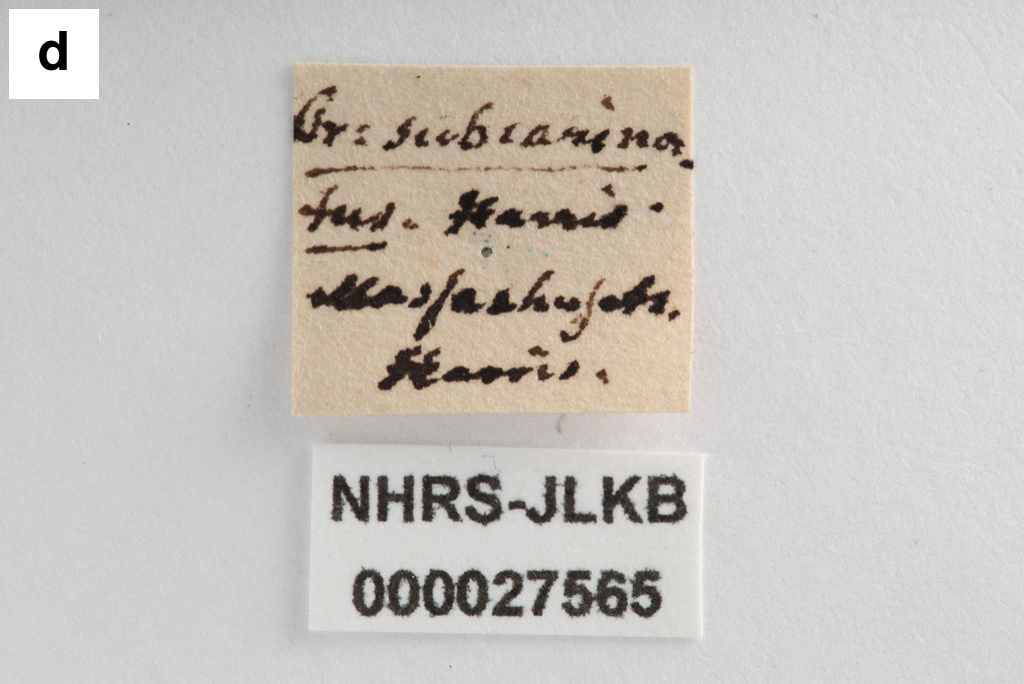
Specimen labels.

**Figure 14a.**
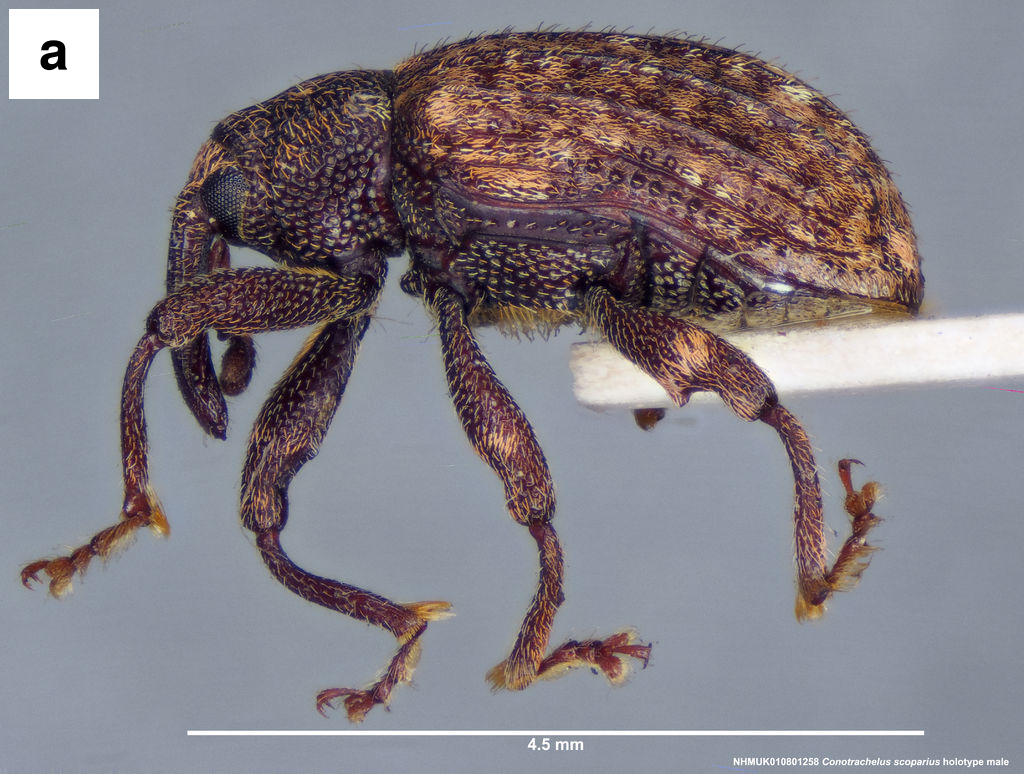
Lateral view.

**Figure 14b.**
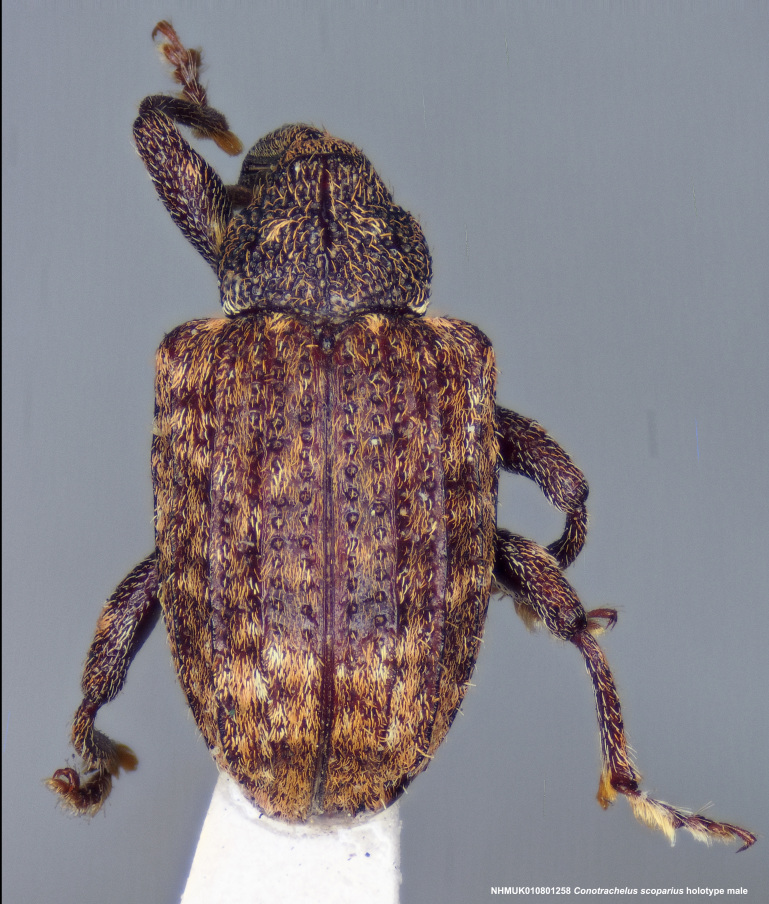
Dorsal view.

**Figure 14c.**
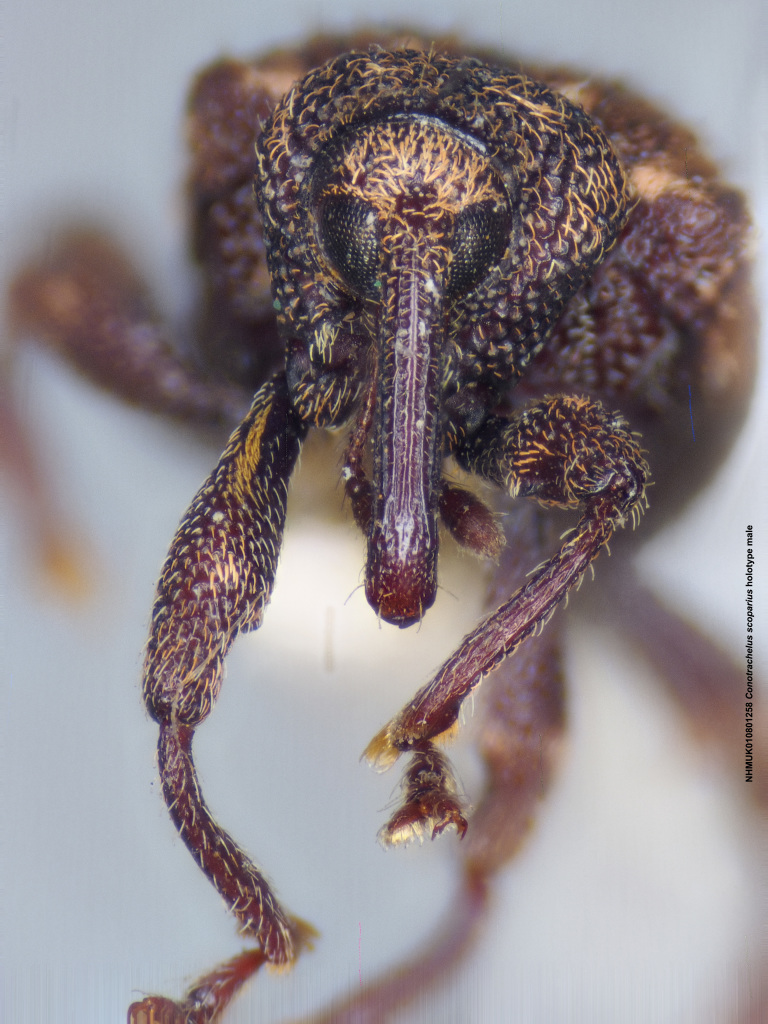
Anterior view.

**Figure 14d.**
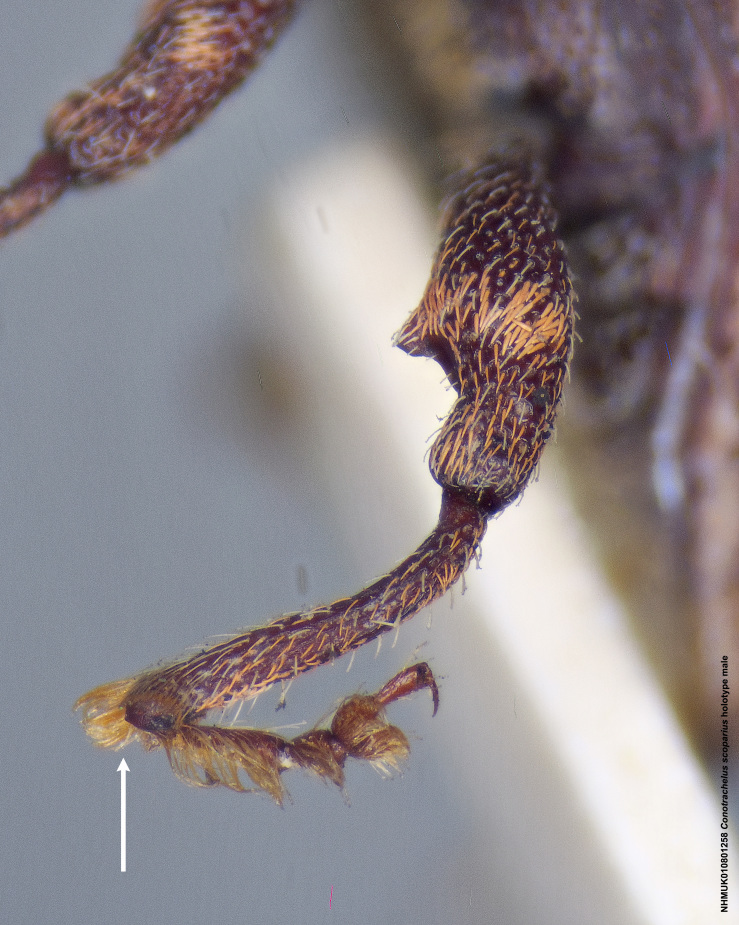
Detail, hind leg, lateral view.

**Figure 14e.**
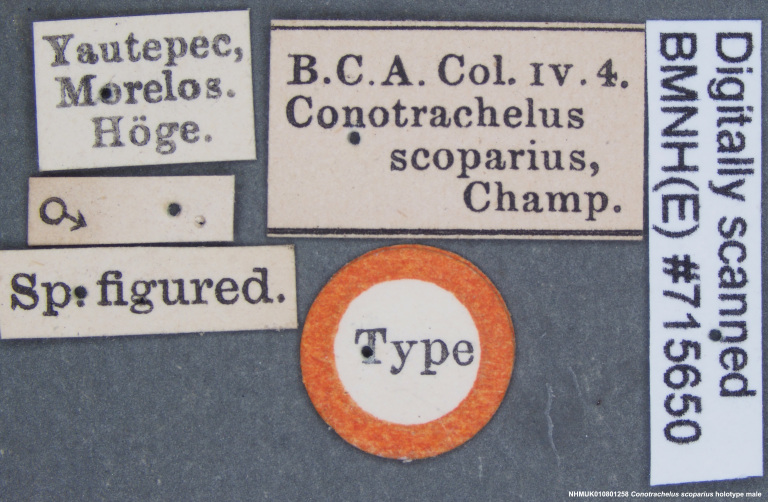
Labels.

**Figure 15a.**
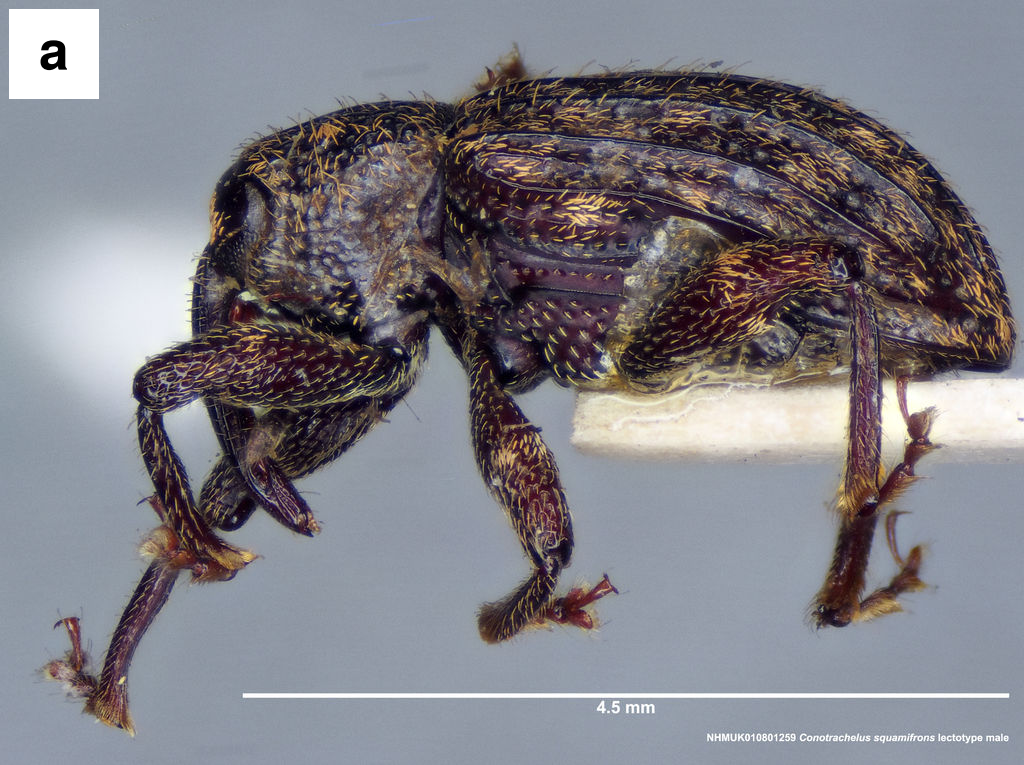
Lateral view.

**Figure 15b.**
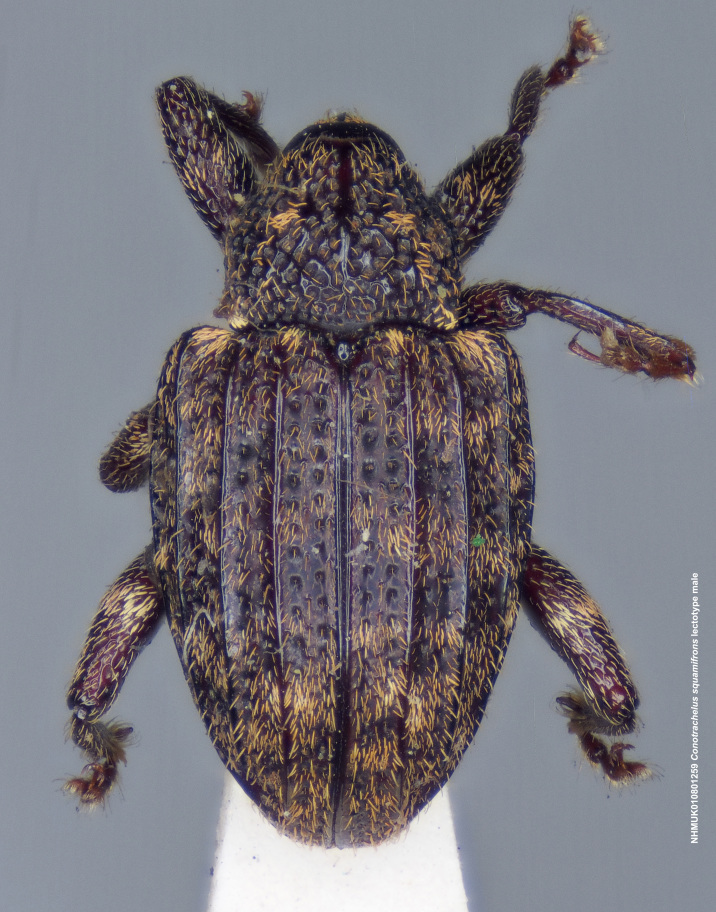
Dorsal view.

**Figure 15c.**
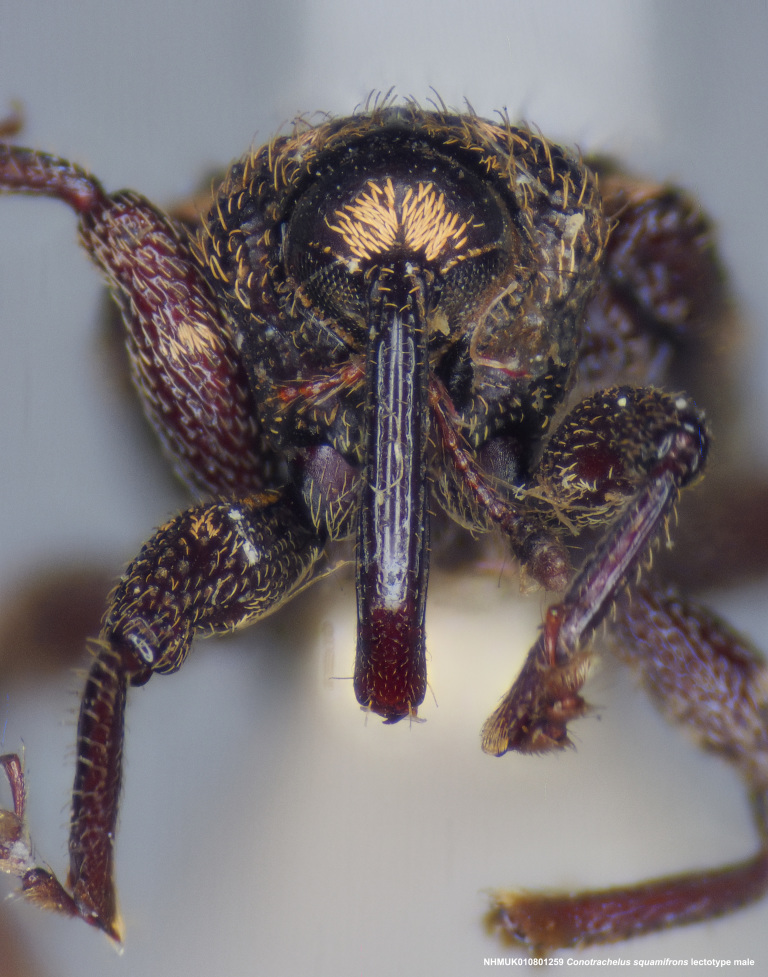
Anterior view.

**Figure 15d.**
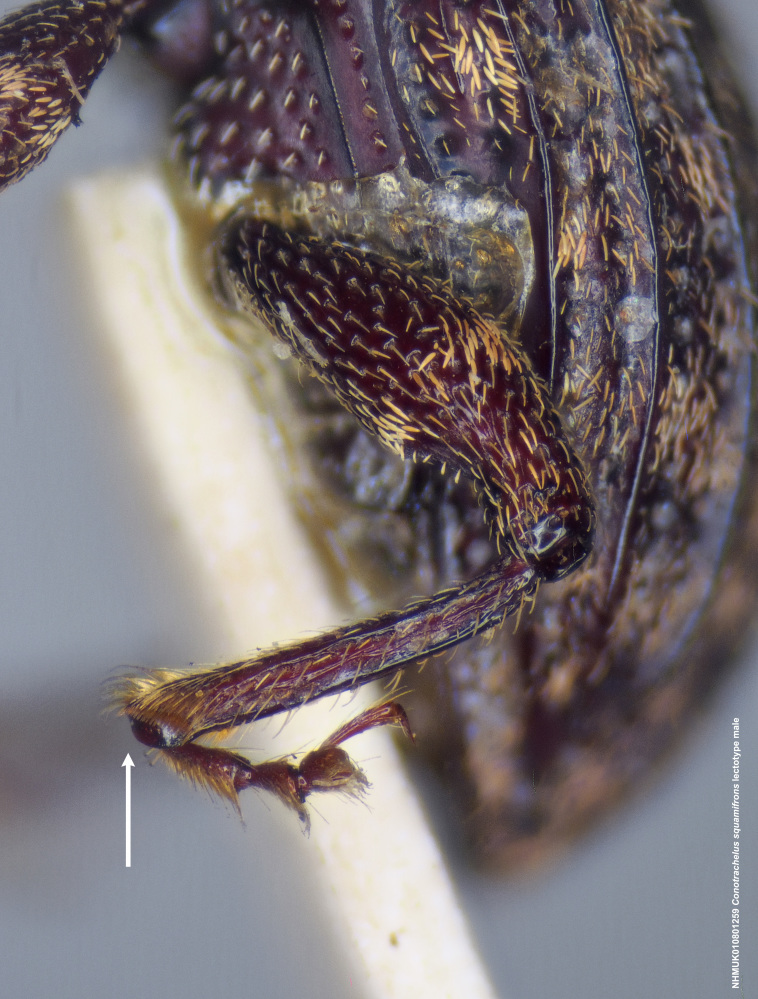
Detail, hind leg, lateral view.

**Figure 15e.**
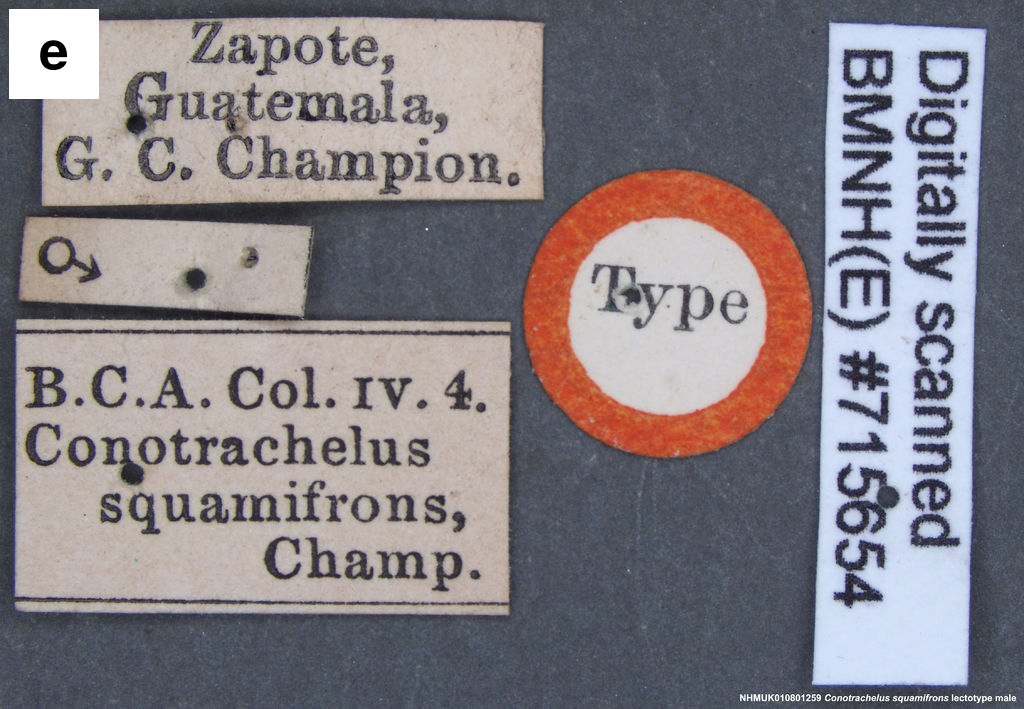
Labels.
